# Integrative alternative splicing analysis reveals new prognosis signature in B-cell acute lymphoblastic leukemia

**DOI:** 10.7150/ijbs.98899

**Published:** 2024-08-19

**Authors:** Zhiyi Zhuo, Junfei Wang, Yonglei Zhang, Guoyu Meng

**Affiliations:** 1 Shanghai Institute of Hematology, State Key Laboratory of Medical Genomics, National Research Center for Translational Medicine, Rui-Jin Hospital, Shanghai Jiao Tong University School of Medicine and School of Life Sciences and Biotechnology, Shanghai Jiao Tong University, 197 Ruijin Er Road, Shanghai 200025, P. R. China.; 2 Department of Geriatrics and Medical Center on Aging, Ruijin Hospital, Shanghai Jiao Tong University School of Medicine, Shanghai 200025, P. R. China.; 3 State Key Laboratory of Pathogenesis, Prevention and Treatment of High Incidence Diseases in Central Asia, First Affiliated Hospital of Xinjiang Medical University, Xinjiang, P. R. China.

**Keywords:** B cell acute lymphoblastic leukemia, alternative splicing, prognosis prediction, machine learning algorithms, bioinformatics

## Abstract

The dysregulation of alternative splicing (AS) is increasingly recognized as a pivotal player in the pathogenesis, progression, and treatment resistance of B-cell acute lymphoblastic leukemia (B-ALL). Despite its significance, the clinical implications of AS events in B-ALL remain largely unexplored. This study developed a prognostic model based on 18 AS events (18-AS), derived from a meticulous integration of bioinformatics methodologies and advanced machine learning algorithms. The 18-AS signature observed in B-ALL distinctly categorized patients into different groups with significant differences in immune infiltration, V(D)J rearrangement, drug sensitivity, and immunotherapy outcomes. Patients classified within the high 18-AS group exhibited lower immune infiltration scores, poorer chemo- and immune-therapy responses, and worse overall survival, underscoring the model's potential in refining therapeutic strategies. To validate the clinical applicability of the 18-AS, we established an SF-AS regulatory network and identified candidate drugs. More importantly, we conducted in vitro cell proliferation assays to confirm our analysis, demonstrating that the High-18AS cell line (SUP-B15) exhibited significantly enhanced sensitivity to Dasatinib, Dovitinib, and Midostaurin compared to the Low-18AS cell line (REH). These findings reveal AS events as novel prognostic biomarkers and therapeutic targets, advancing personalized treatment strategies in B-ALL management.

## Introduction

Acute lymphoblastic leukemia (ALL) is a progressive malignant tumor of the blood, caused by the uncontrolled proliferation of lymphoblasts in hematopoietic and lymphoid tissues, particularly in the bone marrow, spleen, and lymph nodes^1^. ALL is the most common type of leukemia in children, with B-cell acute lymphoblastic leukemia (B-ALL) accounting for 80% of cases. Although over 80% of children with ALL achieve remission, relapse remains a leading cause of pediatric death. In adults, ALL is less common but shows greater biological diversity, clinical heterogeneity, and worse outcomes^2^. Therefore, there is an urgent need to explore new potential molecular mechanisms underlying B-ALL to improve the prognosis of patients with B-ALL.

In recent years, the advancement of high-throughput sequencing technologies has enabled the identification of new molecular biomarkers for the prognosis of B-ALL patients through sequencing data analysis. Prior research has largely focused on gene fusions, gene expression, or gene mutations^3^. However, the low tumor mutational burden (TMB) observed in ALL patients suggests that analyses based solely on gene expression or mutations may not adequately account for the differences in key pathogenic processes^4^. Alterations in the transcriptome and proteome often signal the onset and progression of diseases. Alternative splicing (AS), a principal mechanism regulating the complexity and functional diversity of the transcriptome and proteome in eukaryotes^5^, not only plays a significant role in normal cellular expression regulation and organismal development, such as in hematopoiesis^6^, brain development^7^, and muscle function^8^; it is also intimately associated with the emergence of many systemic diseases and even cancers. In cancer cells, certain crucial genes produce splice isoforms, which differ from those in normal cells, through alternative splicing, thereby directly contributing to tumor development^9^-^11^. Upon analyzing transcriptome/whole-genome data in B-ALL, researchers have identified abnormal alternative splicing events within B-ALL that are associated with its onset, progression^12^, ^13^, drug resistance^14^, and prognosis^15^. For example, previous studies have shown that an alternative splicing transcript of the NT5C2 gene, incorporating an alternative exon 4A, generates an enzyme that deactivates nucleoside analog drugs used in ALL treatment, leading to drug resistance and cancer relapse^16^-^18^. Furthermore, research by Sotillo E et al. revealed that alternative splicing of CD19 and CD22 could lead to loss or modification of chimeric antigen receptor (CAR) T-cell therapy targets, preventing CAR T cells from effectively recognizing and eliminating tumor cells, thereby resulting in acquired immune therapy resistance^14^. Our team has also previously demonstrated that the aberrant expression of DUX4/IGH in B-ALL patients and the subsequent abnormal alternative splicing it drives, namely ERG_alt_, are primary driving factors for the comprehensive onset of leukemia, establishing ERG_alt_ as a significant secondary hit in leukemia development^13^. Therefore, actively understanding the alternative splicing events during the progression of B-ALL is extremely valuable and necessary for the prognosis and clinical treatment of B-ALL.

**Figure 1:**
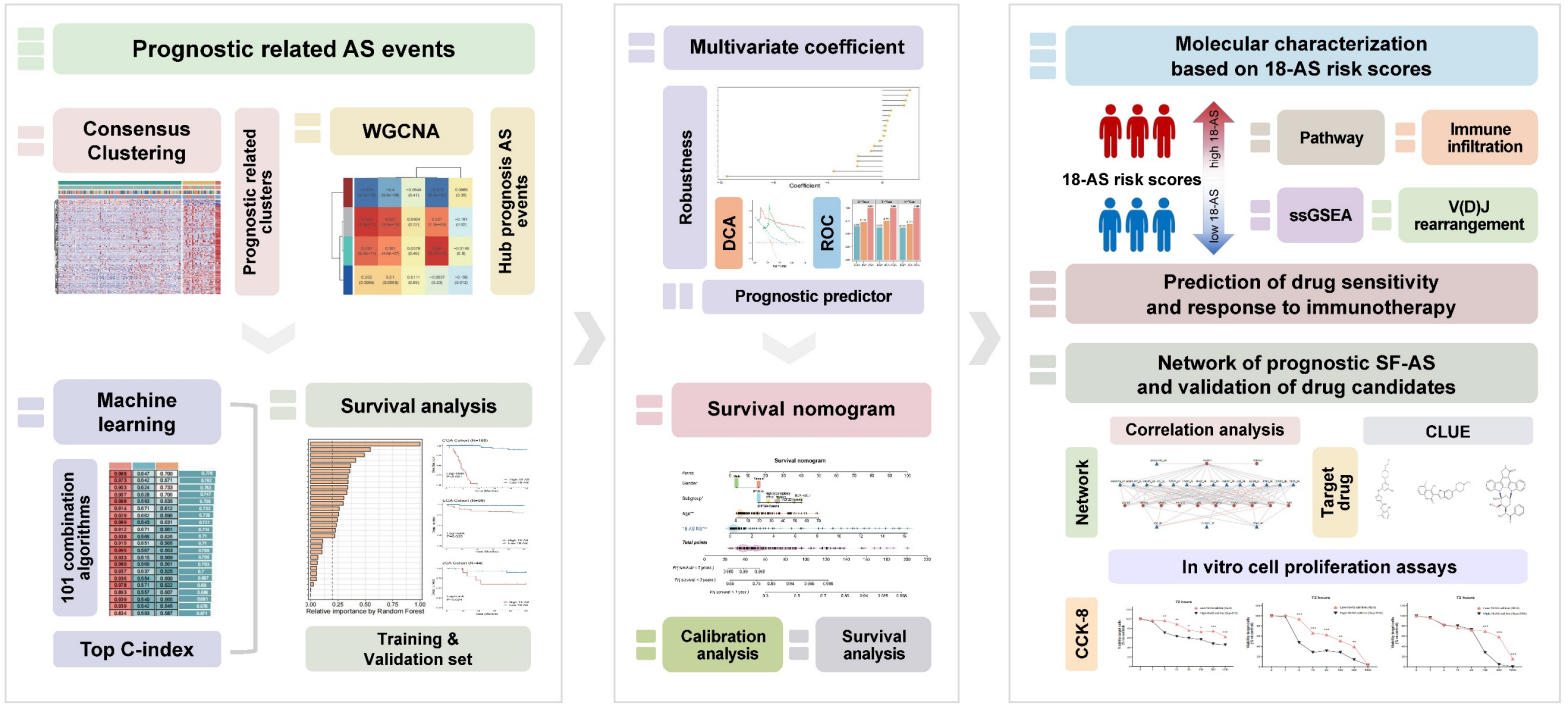
** Workflow of This Study.** This figure presents the study's streamlined approach, starting from data collection to uncovering prognostic insights in B-ALL, mainly including the analysis of RNA-seq data to identify alternative splicing events, the development and validation of a prognostic model using machine learning, and the exploration of immunological and drug response implications.

Previous research on B-ALL has primarily focused on the effects of gene mutations and dysregulation on function and clinical treatment, while the role of alternative splicing events, which directly influence transcript structure, has largely been overlooked. In B-ALL, the potential of AS remains largely untapped. Therefore, in this work, we aimed to develop and validate risk stratification signatures based on prognostically relevant AS events among 303 B-ALL patients from three independent datasets. We employed a range of bioinformatics methods, in conjunction with machine learning models, to comprehensively identify and interpret AS events as prognostic indicators (Fig. 1). Currently, the prognosis of B-ALL is primarily based on classifications involving fusion genes and chromosomal abnormalities. Our work introduces a novel perspective for the clinical diagnosis and treatment of B-ALL, potentially contributing to the optimization of precision therapies for B-ALL and further improving patient outcomes.

## Methods

### Data Collection and Preprocessing

This study received approval from the Ethics Committee of Ruijin Hospital. The CGA cohort (N = 165, training set) RNA-seq raw data and clinical data (Table S1) were obtained through the hospital network database of the Shanghai Institute of Hematology (SIH). The B-ALL patients were enrolled under the Shanghai Institute Hematology protocol (Chinese Clinical Trial Registry, number ChiCTR-RNC-14004969) and Shanghai Children's Medical Center protocol (Chinese Clinical Trial Registry, number ChiCTR-ONC-14005003). The EGA cohort (N = 95, validation set) RNA-seq raw data were accessed through the European Genome-phenome Archive (accession number EGAS00001001795), and the JGA cohort (N = 44, validation set) RNA-seq raw data were obtained from the Japanese Genotype-Phenotype Archive (accession number JGAS00000000047). The transcriptome data of all patients analyzed in this study had previously been analyzed as part of prior publications^19^-^22^.

RNA-seq data were aligned with the human reference genome (GRCh38) for RNA-seq read mapping, using Salmon (v 0.13.1) to obtain transcript reads. Detailed procedures of quality assessment, reading pair alignment from RNA-seq data are listed in Supplemental Methods.

### Detection of alternative splicing events and screening of high-confidence events

Alternative Splicing events were analyzed using SUPPA2 (v 2.3)^23^, which can identify seven types of AS events: exon skipping (ES), retained intron (RI), alternative 5' splice-site (A5), alternative 3' splice-site (A3), alternative first exons (AF), alternative last exons (AL), and mutually exclusive exons (ME), and explicitly calculates the percent spliced in (PSI) values for splicing events. Detailed procedures are listed in Supplemental Methods.

The resulting raw PSI matrix was filtered using the following logic to obtain high-confidence AS events: i) AS events with less than 20% missing PSI values; ii) AS events with a mean PSI value of > 0.05 and < 0.95; iii) AS events with a PSI variance > 0.1.

### Identification of Candidate Prognostic AS Events and Consensus Clustering

The survival R package was utilized to conduct univariate Cox regression analysis on the association between AS events and patients' overall survival time. Candidate prognostic AS events were those with a* P*-value of < 0.001. A resampling-based consensus clustering method was applied to cluster candidate prognostic AS events in the CGA cohort, performed by the ConsensusClusterPlus R package^24^. The optimal number of clusters was determined by integrating the consensus score matrix, Cumulative Distribution Function (CDF) curves, and Proportion of Ambiguous Clustering (PAC) scores.

### Weighted Gene Co-expression Network Analysis (WGCNA)

The WGCNA R package^25^ was employed to generate a network of co-expressed AS event PSI profiles for the CGA cohort. An appropriate soft threshold β was calculated to satisfy the criteria for a scale-free network. The dynamic tree cutting method was used for module identification. To identify AS event modules significantly related to the consensus clusters, the module with the highest correlation was selected for further study.

### Prognostic AS Signatures Identified Through an Ensemble Machine Learning pipeline

To characterize prognostic AS tags with high precision and stability, we integrated ten machine learning algorithms and 101 algorithm combinations based on previous research^26^. The ensemble algorithms included Random Survival Forest (RSF), Elastic Net (Enet), Lasso, Ridge, Step Cox, CoxBoost, Cox Partial Least Squares Regression (plsRcox), Supervised Principal Component (SuperPC), Generalized Boosted Regression Modeling (GBM), and Survival Support Vector Machine (survival-SVM). Key prognostic AS events from the module with the highest correlation to consensus clusters obtained from the WGCNA procedure were fitted using 101 algorithm combinations within a predictive model frameworkin the CGA cohort. All models were validated in two validation datasets, EGA and JGA. For each model, the Harrell's concordance index (C-index) was calculated across all validation datasets, and the model with the highest average C-index was considered optimal.

### Estimation of Immune Cell Infiltration in the Tumor Microenvironment

The estimation of immune cell proportions in patients was conducted using seven algorithms implemented by the R package IOBR^27^. These algorithms include Cibersort, TIMER, quanTIseq, MCP-counter, xCell, EPIC, and ESTIMATE, which infer the proportions of immune cells in patients based on the expression of immune cell marker genes in samples.

### Immunoglobulin Repertoire V(D)J Rearrangement Analysis

After quality assessment of the RNA-seq data, immunoglobulin repertoire information was extracted using MiXCR (v4.0)^28^. The Convert program of VDJtools (v 1.2.1)^29^ was used for format conversion, and the PlotFancyVJUsage and RarefactionPlot programs were utilized for visualization. Detailed procedures are listed in Supplemental Methods.

### Prediction of Drug Sensitivity and Evaluation of Immune Therapy Response

The oncoPredict R package^30^ was used to predict the half-maximal inhibitory concentration (IC_50_) of drugs commonly used for treating ALL in each sample. The TIDE algorithm (http://tide.dfci.harvard.edu/login/)^31^ was employed to assess the potential clinical efficacy of immunotherapy across different groups, reflecting the potential for tumor immune evasion. A higher TIDE score is associated with poorer efficacy of immune checkpoint inhibitors (ICIs).

### SF-AS Regulatory Network

A list of splicing factors was collected from previous research^32^. Spearman correlation analysis was utilized to analyze the correlation between the PSI values of prognostic AS events and the expression of SFs. SF-AS relationships with a p-value less than 0.05 and an absolute Spearman correlation coefficient greater than 0.25 were selected, and an SF-AS regulatory network was constructed using Cytoscape^33^.

### Cell Proliferation Analyses

10,000 REH or Sup-B15 cells were seeded into each well of a 96-well plate. The cells were treated with Dasatinib, Dovitinib, or Midostaurin at concentrations ranging from 1 to 1000 nM. They were cultured in RPMI 1640 medium supplemented with 10% FBS, with a total volume of 100 μl per well, and incubated at 37°C in a humidified atmosphere containing 5% CO2 for 72 hours. After the incubation period, 10 μl of Cell Counting Kit-8 (CCK-8, Vazyme) reagent was added to each well and the plates were further incubated for 2 hours at 37°C. Absorbance at 450 nm was then measured using a microplate reader to determine cell viability based on the absorbance values.

### RT-PCR

SUP-B15 (High-18-AS) and REH (Low-18-AS) leukemia cell lines were cultured. Total RNA was extracted using the RNeasy Mini Kit (Vazyme, Nanjing, China). The synthesis of the first-strand complementary DNA was accomplished using the HiScript II RT SuperMix II Kit (Vazyme, Nanjing, China). Subsequently, primers were designed based on the reverse-transcribed first-strand (Table S3), and Polymerase chain reactions (PCR) were carried out using the Taq Plus Master Mix (Vazyme, Nanjing, China). The program of PCR was as follows: 95°C, 5 min to activateadvantage GC polymerase; followed by 35 cycles of 94°C, 30 s; 55°C, 30 s and 72°C for 45 s, and finalextension was performed at 72°C for 5 min. After obtaining the PCR products, electrophoresis was performed using 0.7% agarose gel.

### Statistical Analysis

All downstream data processing, statistical analyses, and plotting were conducted in R software version 4.1.3. The correlation between two continuous variables was assessed using Pearson or Spearman correlation coefficients. Categorical variables were compared using the Wilcoxon rank-sum test, while continuous variables were compared using the Wilcoxon rank-sum test or T-test as appropriate. Cox regression and Kaplan-Meier analyses were performed using the survival R package. The time-dependent area under the ROC curve (AUC) for survival variables was conducted by the timeROC R package. DCA curves were plotted using the R package ggDCA^34^. Forest plots were generated with ggforest in the survminer package. Other R packages used for visualization include pheatmap, clusterProfiler^35^, ggplot2, ggsignif, ggsankey, ggpubr, factoextra, gghalves, tinyarray, and linkET.

## Results

### Selection of Key Prognostic-Related AS Events

**Figure 2:**
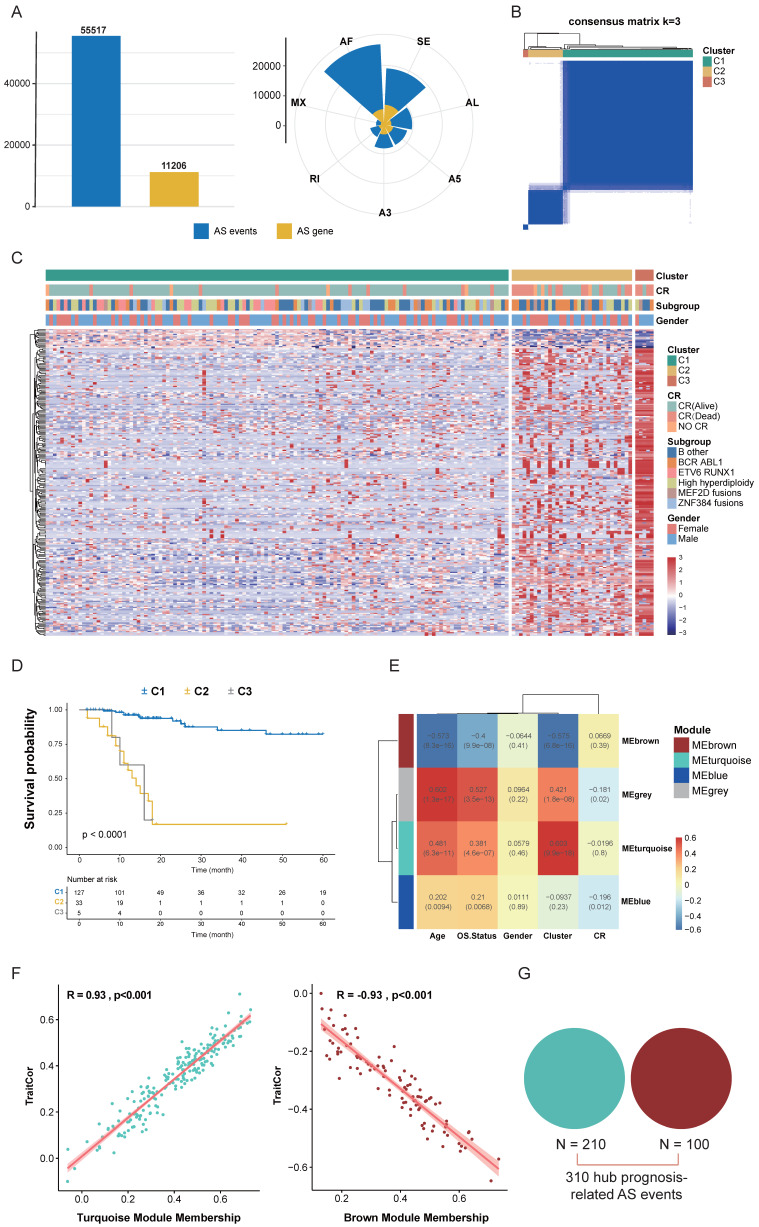
** Identification of Key Prognostic-Related AS Events.** (A) Distribution of high-confidence AS events and genes undergoing AS across seven types of AS events after filtering. Blue represents AS events, while yellow indicates genes undergoing AS. (B) Consensus heatmap for all samples when k = 3. A higher consensus score between samples indicates a higher likelihood of being grouped into the same cluster across different iterations. (C) Heatmap of the PSI values matrix for AS events across the three consensus clusters C1, C2, C3. (D) Kaplan-Meier curves illustrating survival differences among the three consensus clusters C1, C2, C3. (E) Heatmap showing the correlation between module characteristic AS events and clinical traits. (F) Correlation of module-trait relationships between turquoise and brown modules and consensus clusters, highlighting their significant association. (G) Union of AS events from the turquoise and brown modules identified as key prognostic-related AS events.

To systematically identify prognostic-related alternative splicing events in B-ALL, we first defined the landscape of AS events in B-ALL using RNA-seq data from 165 Chinese B-ALL patients in the Chinese Genome-phenome Archive (CGA) dataset. Overall, we identified over 290,000 distinct AS events in 165 B-ALL patients, including exon skipping (SE), retained intron (RI), alternative 5' splice-site (A5), alternative 3' splice-site (A3), alternative first exons (AF), alternative last exons (AL), and mutually exclusive exons (ME), covering seven types of alternative splicing events. We also observed that a single gene could produce multiple types of AS events (Fig. 2A), confirming the significance of AS in diversifying the B-ALL transcriptome. After data filtering, a final set of 55,517 high-confidence AS events was generated (Fig. 2A). In the subsequent study, we focused on the prognostic value of these high-confidence AS events.

Next, we integrated clinical data from CGA patients and performed univariate Cox analysis on high-confidence AS events, identifying 1,271 screened candidate prognostic AS events (*p* < 0.001). These 1,271 AS events were subjected to Consensus Clustering, initially dividing all B-ALL patient samples into k (k = 2-5) clusters. The Cumulative Distribution Function (CDF) curve of the consensus score matrix and the proportion of the Proportion of Ambiguous Clustering (PAC) statistic indicated that the optimal number of clusters was obtained at k=3 (Fig. 2B, S1A, B). The prognostic differences between the three consensus clusters were significant, with C1 (n = 127) having the best prognosis, followed by C2 (n = 33), and C3 (n = 5) having the worst prognosis (Fig. 2C, D). We then performed WGCNA on the initially screened significant prognostic AS events, setting the soft threshold β to 9 in the WGCNA procedure (Fig. S1C). This provided an appropriate power value for co-expression network construction and identified four modules of different colors (Fig. S1D), showing the correlation between modules and clinical traits such as age, gender, complete remission (CR) status, survival status, and consensus clusters. The turquoise and brown modules had the highest correlation with the consensus clusters (Fig. 2E), both reaching 0.93, indicating good module construction quality (Fig. 2F). To further explore key AS events highly related to the consensus clusters within the turquoise and brown modules, we took the union of AS events within both modules, ultimately considering 310 AS events as key prognostic-related AS events (Fig. 2G).

### Identification of Prognostic AS Signatures Based on an Ensemble Machine Learning Pipeline

**Figure 3:**
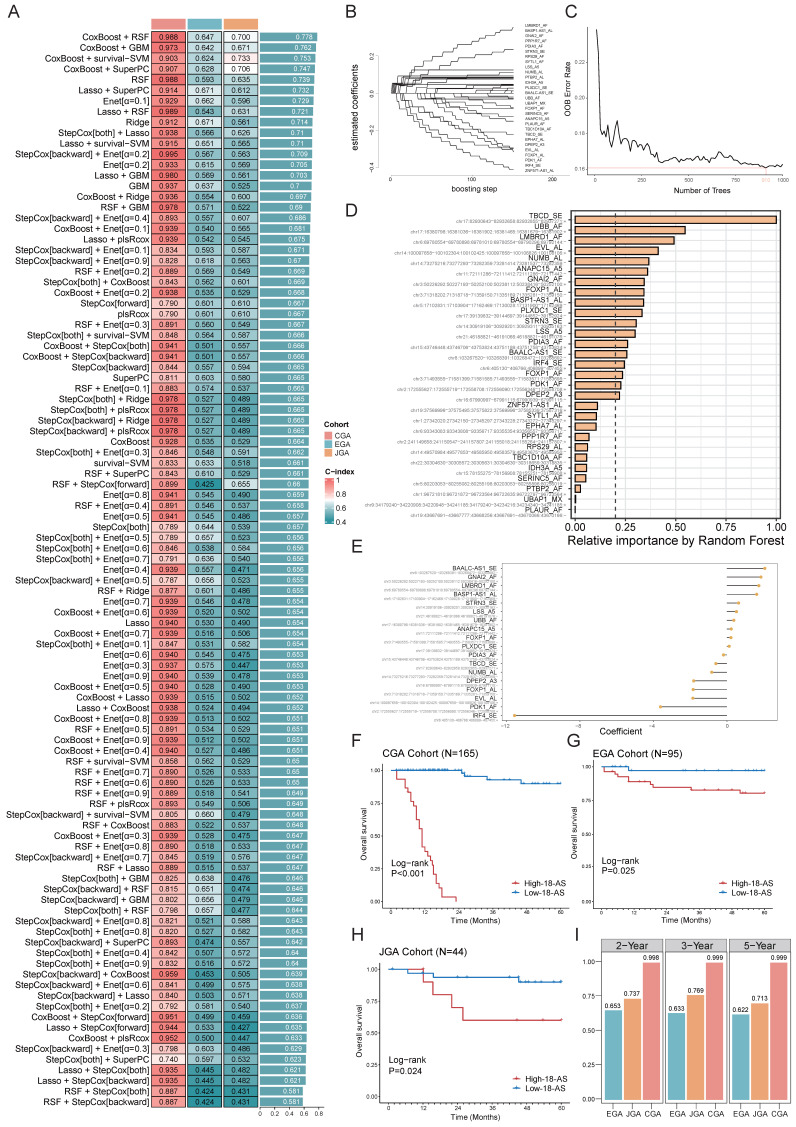
** Identification of Prognostic AS Signatures Using an Ensemble Machine Learning Pipeline.** (A) Prediction across 101 predictive models was conducted using an ensemble machine learning algorithm framework, with further calculation of the C-index for each model across all validation datasets, showcasing the comprehensive evaluation of model performance. (B) The CoxBoost algorithm, after 151 steps, identified 29 AS events with non-zero coefficients, illustrating the rigorous selection process for AS events with significant prognostic impact. (C) Error rate curve of the RSF algorithm, achieving the minimum error rate at n = 910L, highlighting the optimization process for model accuracy. (D) Relative importance ranking of AS events in the RSF algorithm, emphasizing the contribution of individual AS events to the model's predictive power. (E) Final coefficients for 18 AS events obtained through Cox regression, underlining the critical AS events that constitute the prognostic signature. (F-H) Impact of the composite feature comprising 18 AS events on prognosis across CGA (F), EGA(G) and JGA (H) datasets, with Kaplan-Meier survival analysis for B-ALL patients demonstrating the prognostic value of the 18-AS. (I) Time-dependent ROC analysis predicting 2-year, 3-year, and 5-year overall survival (OS) across three datasets, validating the prognostic accuracy of the 18-AS over time.

Next, we aimed to develop prognostic AS signatures based on 310 key prognostic-related AS events through an integration process utilizing machine learning. In the CGA dataset, we fitted 101 predictive models using a machine learning framework and further calculated the concordance index (C-index) of each model across all validation datasets (Fig. 3A). The optimal model was determined to be a combination of CoxBoost and Random Survival Forest (RSF), which achieved the highest average C-index (0.778), and this combination model exhibited significant C-index values across all validation datasets (Fig. 3A). The CoxBoost model, after 151 steps, identified 29 AS events with non-zero coefficient values (Fig. 3B). We further analyzed these 29 AS events using the RSF model, which achieved the minimum error at n = 910, and ranked the 29 AS events by relative importance, selecting those with a relative ranking greater than 0.25. Ultimately, 18 prognostic AS tags were identified (Fig. 3C, D), hereafter referred to as 18-AS signature. Subsequently, each patient's risk score was calculated using the percent spliced in (PSI) values of the 18-AS and their regression coefficients in a Cox model (Fig. 3E). To assess the prognostic significance of 18-AS, we determined the optimal cutoff value using the survminer package, dividing all patients into high 18-AS and low 18-AS groups. Kaplan-Meier survival analysis further demonstrated that patients in the high 18-AS group from the CGA training set had a significantly higher risk of death compared to the low 18-AS group (*p* < 0.001) (Fig. 3F). The same outcome was observed in two validation cohorts from the Japanese and European B-ALL patients from Japanese Genome-phenome Archive (JGA) and European Genome-phenome Archive (EGA) datasets, where the overall survival (OS) of high 18-AS patients was significantly lower than that of the low 18-AS group (*p* < 0.05).

Additionally, we measured the discriminative ability of 18-AS using time-dependent receiver operating characteristic (Time ROC) analysis, with the 2-year, 3-year, and 5-year area under the curve (AUC) for CGA patients being 0.998, 0.999, and 0.999, respectively. For JGA patients, the AUCs were 0.737, 0.769, and 0.713, and for EGA patients, they were 0.653, 0.633, and 0.622, respectively (Fig. 3I). Furthermore, decision curve analysis (DCA) indicated that 18-AS provided a greater net survival benefit than other markers in the CGA and JGA datasets (Fig. S2). To facilitate risk quantification, we constructed a nomogram (Fig. S2) that combines 18-AS with clinical information, where higher nomogram points indicate poorer patient prognosis. These results suggest that prognostic AS signatures exhibit superior performance across each dataset, highlighting the broad clinical application prospects of AS events in B-ALL patients (Fig. S3).

**Figure 4:**
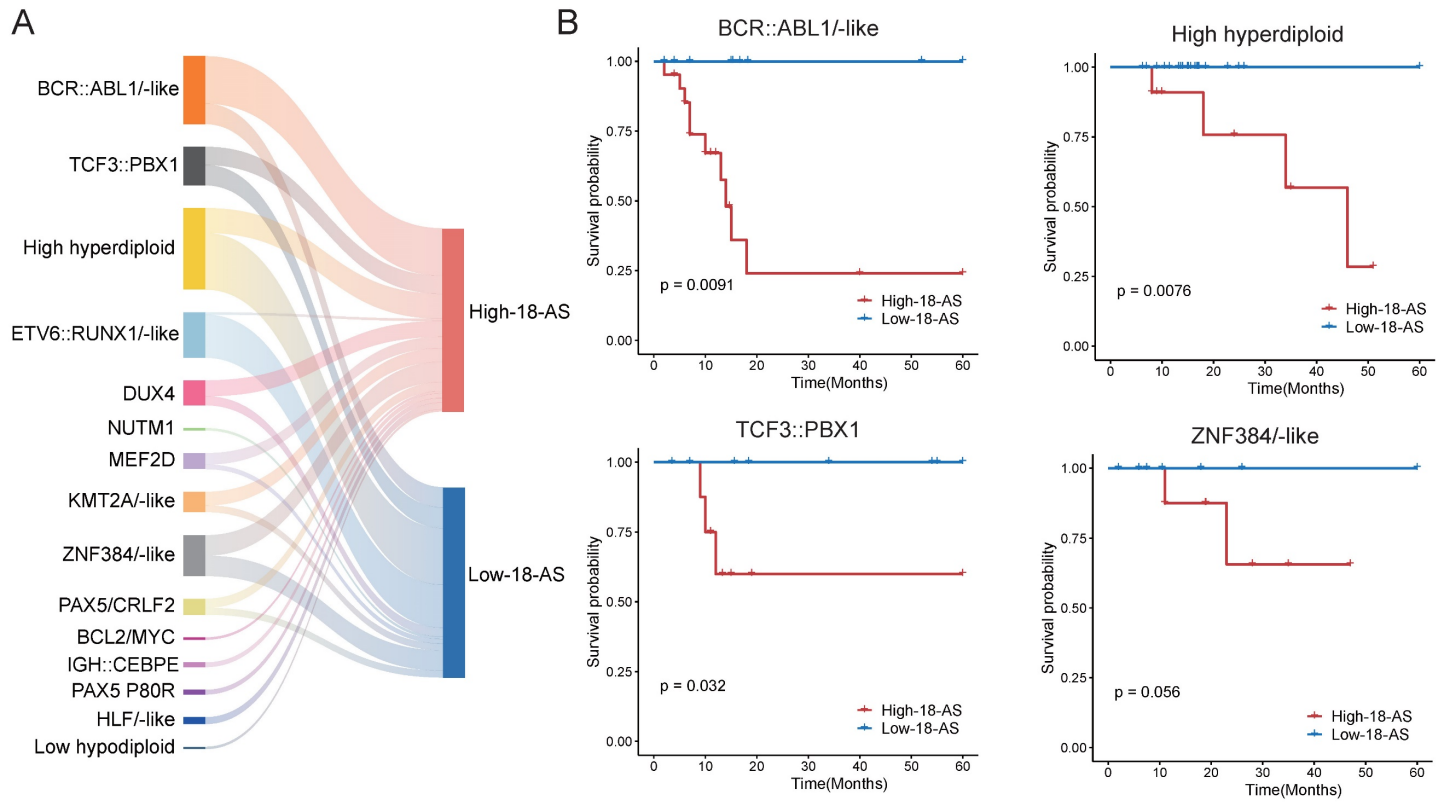
** Prognostic Value of the 18-AS Across Different B-ALL Subtypes.** (A) Mulberry chart of patient subtype distribution based on 18-AS, where the width of the bars is proportional to the quantity ratio. (B) Kaplan-Meier survival curves for patients with the different subtype stratified by high-18-AS and low-18-AS groups.

To further validate the prognostic value of the 18-AS across different B-ALL subtypes, our results showed that the 18-AS can significantly distinguish high-risk from low-risk patients within multiple known B-ALL subtypes (Fig. 4). For instance, in the BCR::ABL1/-like subtype, patients in the high 18-AS group had significantly lower survival rates compared to those in the low 18-AS group (*p* = 0.0091). Similar stratification effects were observed in other subtypes such as high hyperdiploid (*p* = 0.0076), TCF3::PBX1(*p* = 0.032), and ZNF384/-like subtypes (*p* = 0.056), indicating the broad prognostic applicability of the 18-AS.

Moreover, we performed survival analyses separately for pediatric (aged under 18 years) and adult (aged 18 years and older) patients to address the concern that the 18-AS might merely reflect age-related prognostic differences (Fig. S4). The results demonstrated that the 18-AS can significantly stratify survival outcomes in both pediatric and adult patients (*p* < 0.001), underscoring its independent prognostic value across different age groups.

### Differences in Immunological and Molecular Characteristics Based on 18-AS

**Figure 5:**
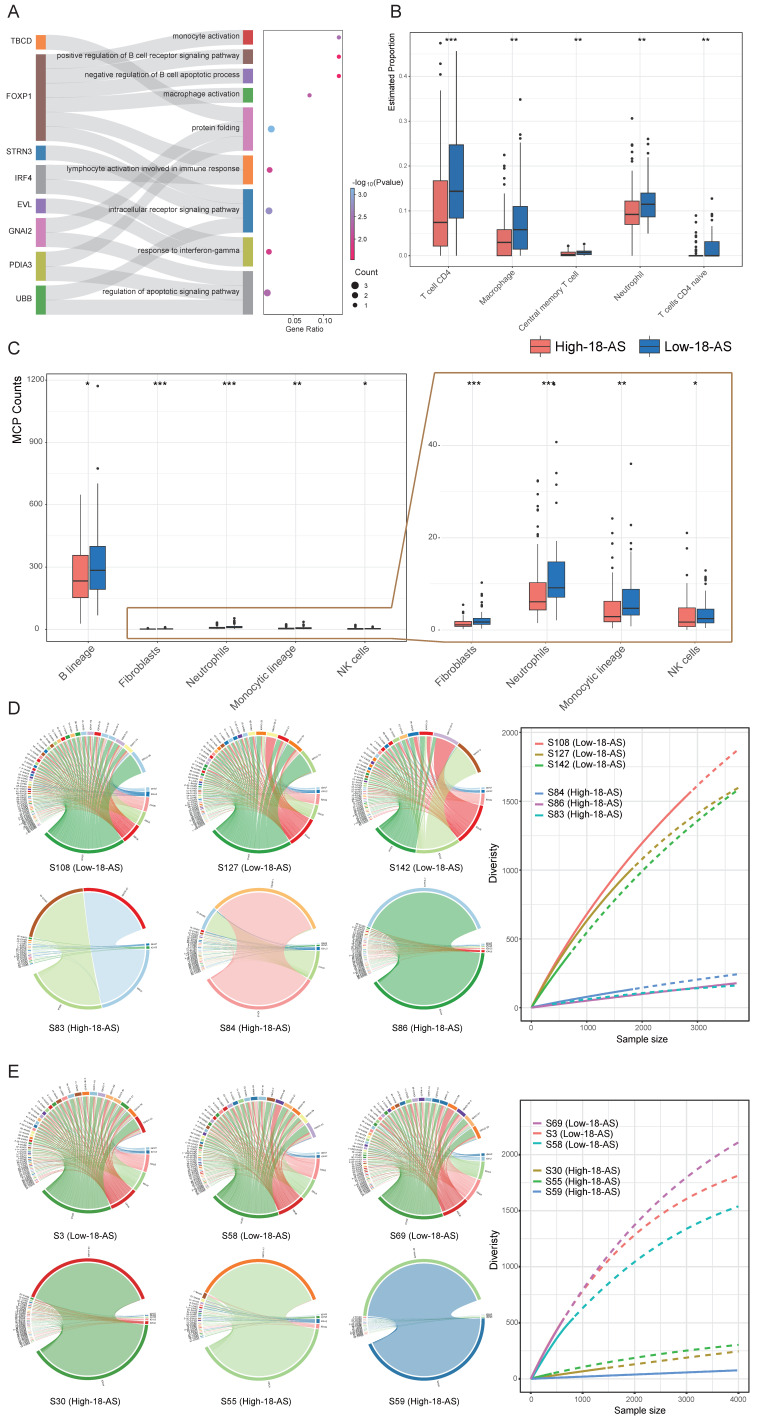
** Differences in Immunological and Molecular Characteristics Based on 18-AS.** (A) Enrichment analysis of 18-AS genes, displaying genes undergoing AS events (left panel) and corresponding pathways (right panel). A mulberry chart shows the distribution of genes involved in pathways, illustrating the intricate network of AS events and their pathway implications. (B-C) Distribution of immune cell subpopulation infiltration across different 18-AS subgroups, revealing the impact of AS events on the tumor microenvironment and immune cell dynamics. Differences in fibroblasts, neutrophils, monocytic lineage and NK cells were shown in enlarged view on the far right panel. (D) Circos plot of V_H_J_H_ rearrangement in pediatric patients across different 18-AS subgroups, where the width of the bands correlates with the frequency of rearrangement events. The diversity of V_H_J_H_ rearrangements in patients with different 18-AS subgroups is summarized in the curve plot on the right. (E) Circos plot of V_H_J_H_ rearrangement in adult patients across different 18-AS subgroups, similarly illustrating the proportional relationship between band width and rearrangement event frequency. The diversity of V_H_J_H_ rearrangements in patients with different 18-AS subgroups is summarized in the curve plot on the right. Statistical comparisons of categorical variables were made using the Wilcoxon rank-sum test, with significance levels marked as *, *P* < 0.05; **, *P* < 0.01; ***, *P* < 0.001.

Patients in the high 18-AS group exhibited a significantly higher risk of death compared to those in the low 18-AS group, consistent with the clinical characteristics of B-ALL observed in our study. Subsequently, we further evaluated the impact of 18-AS on the patient's immune microenvironment and the abundance of B-cell lineage. We first conducted enrichment analysis on the genes of 18-AS, including genes frequently reported in hematological tumors such as FOXP1, IRF4, PDK1, *etc*. The results showed that these genes were enriched in pathways such as monocyte activation, macrophage activation, B-cell receptor signaling, B-cell apoptosis, and lymphocyte activation involved in the immune response (Fig. 5A). Meanwhile, patients with different 18-AS subgroups showed significant differences in immune infiltration, with high 18-AS patients having lower abundance of B cells, CD4 T cells, macrophages, central memory T cells, monocytes, and NK cells compared to low 18-AS patients (Fig. 5B, C).

Immunoglobulin (Ig) genes are assembled from variable (V), diversity (D), and joining (J) gene segments, undergoing site-specific DNA rearrangement mechanisms of V(D)J recombination during early B-cell differentiation, which determines antigen receptor diversity^36^. In both pediatric and adult high 18-AS patients, we observed defects in V(D)J rearrangement (Fig. 5D, E, S5). Compared to the high V(D)J recombination diversity in low 18-AS patients, high 18-AS patients exhibited a more pronounced oligoclonal state, showing significant reductions in V_H_J_H_ and V_k_J_k_ rearrangements. This may suggest that malfunction in RNA splicing in the context of high 18-AS patients could cause more severe impairment in the proliferation, survival, and differentiation of precursor B cells at an early stage, resulting in poor drug response.

### Drug Sensitivity and Immune Response Prediction Based on 18-AS

**Figure 6:**
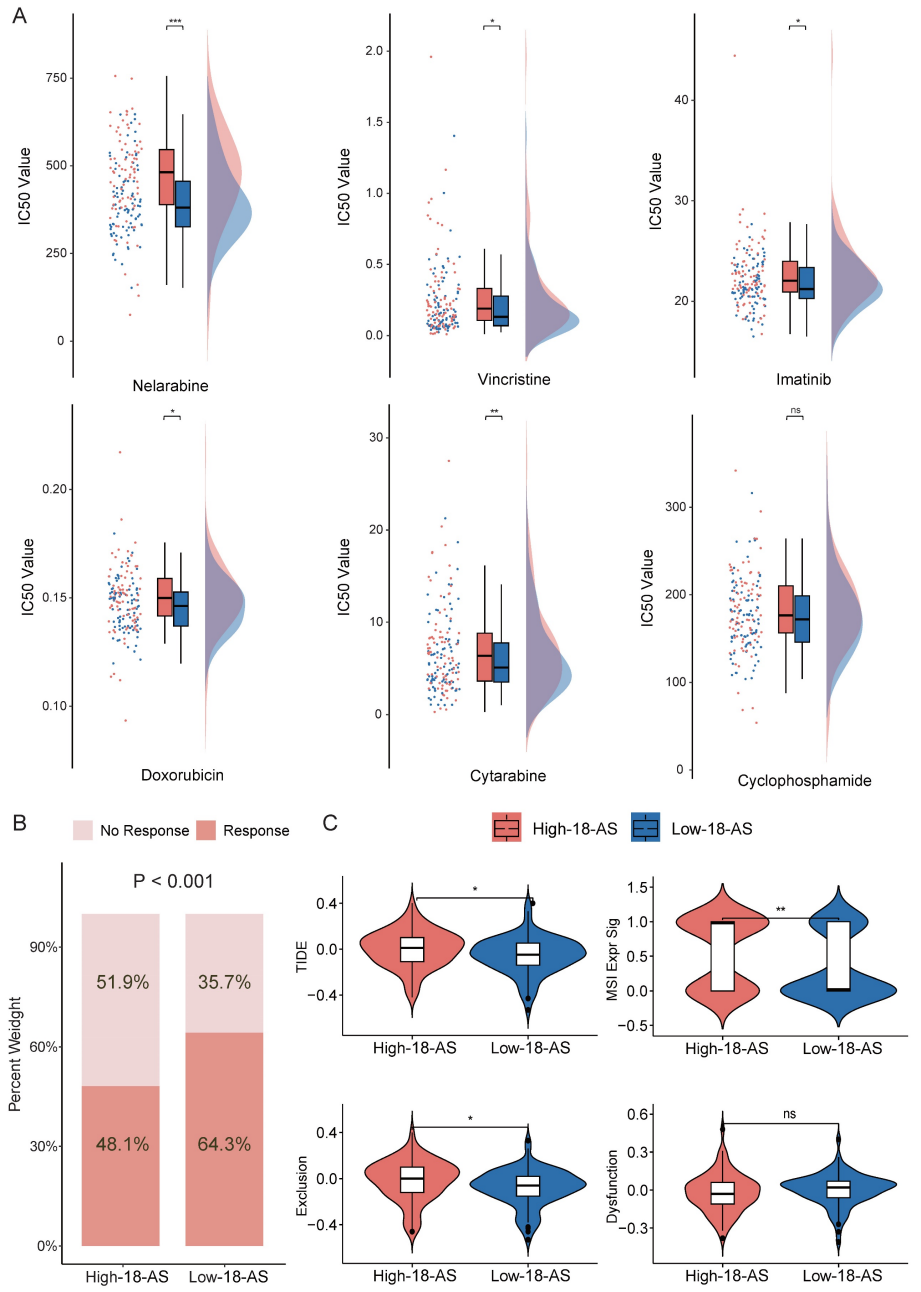
** Drug Sensitivity and Immune Response Prediction Based on 18-AS.** (A) Comparison of the predicted IC_50_ value distributions for six drugs between 18-AS subgroups. The IC_50_ value indicates the effectiveness of a substance in inhibiting a specific biological or biochemical function, with smaller values indicating better efficacy. (B) Differences in immune therapy responses between 18-AS subgroups based on the TIDE algorithm. (C) TIDE scores for 18-AS subgroups. TIDE scores correlate positively with the tumor's potential for immune evasion. The Exclusion score correlates positively with the expression of T-cell exclusion markers, the Dysfunction score correlates positively with the expression of T-cell dysfunction markers, and the MSI score negatively correlates with microsatellite instability. Categorical variables were compared using the Wilcoxon rank-sum test, and continuous variables were compared using the T-test, with significance levels indicated as *, *P* < 0.05; **, *P* < 0.01; ***, *P* < 0.001.

To investigate the clinical therapeutic value of the 18-AS prognostic model, we explored differences in the efficacy of chemotherapy and immunotherapy among patients with different 18-AS subgroups. We referenced the list of FDA-approved drugs (https://www.cancer.gov/about-cancer/treatment/drugs/leukemia#1) for treating ALL and, in conjunction with the actual medication usage in the CGA cohort of Chinese patients (Chinese Clinical Trial Registry; no. ONC-14004969, ONC-14005003), conducted drug sensitivity analysis using oncoPredict package. Among six common chemotherapy drugs used to treat B-ALL, patients in the high 18-AS group exhibited higher IC_50_ values compared to those in the low 18-AS group (Fig. 6A), with significant differences in the IC_50_ values for Nelarabine, Vincristine, Imatinib, Doxorubicin, and Cytarabine (*p* < 0.05). Overall, high 18-AS patients displayed significant reduced sensitivity to these drugs, reiterating their poor response to chemo- and targeted-therapies.

With increasing evidence showing that chemotherapy/targeted therapy combined with immune checkpoint therapy can demonstrate better efficacy in leukemia treatment^37^, ^38^, we predicted immune therapy responses in patients. We observed significantly lower immune therapy responses in high 18-AS patients (48.1%) compared to low 18-AS patients (64.3%, *p* < 0.001, Fig. 6B), with high 18-AS patients having higher TIDE scores and T-cell dysfunction scores (Fig. 6B). These findings suggest a lower efficiency of immune therapy in high 18-AS patients relative to those in the low 18-AS group. Furthermore, the low 18-AS group exhibited higher microsatellite instability (MSI) (Fig. 6C), which is often associated with better prognosis and sensitivity to checkpoint immunotherapy.

### Construction of a Regulatory Network between 18-AS and Splicing Factors

**Figure 7:**
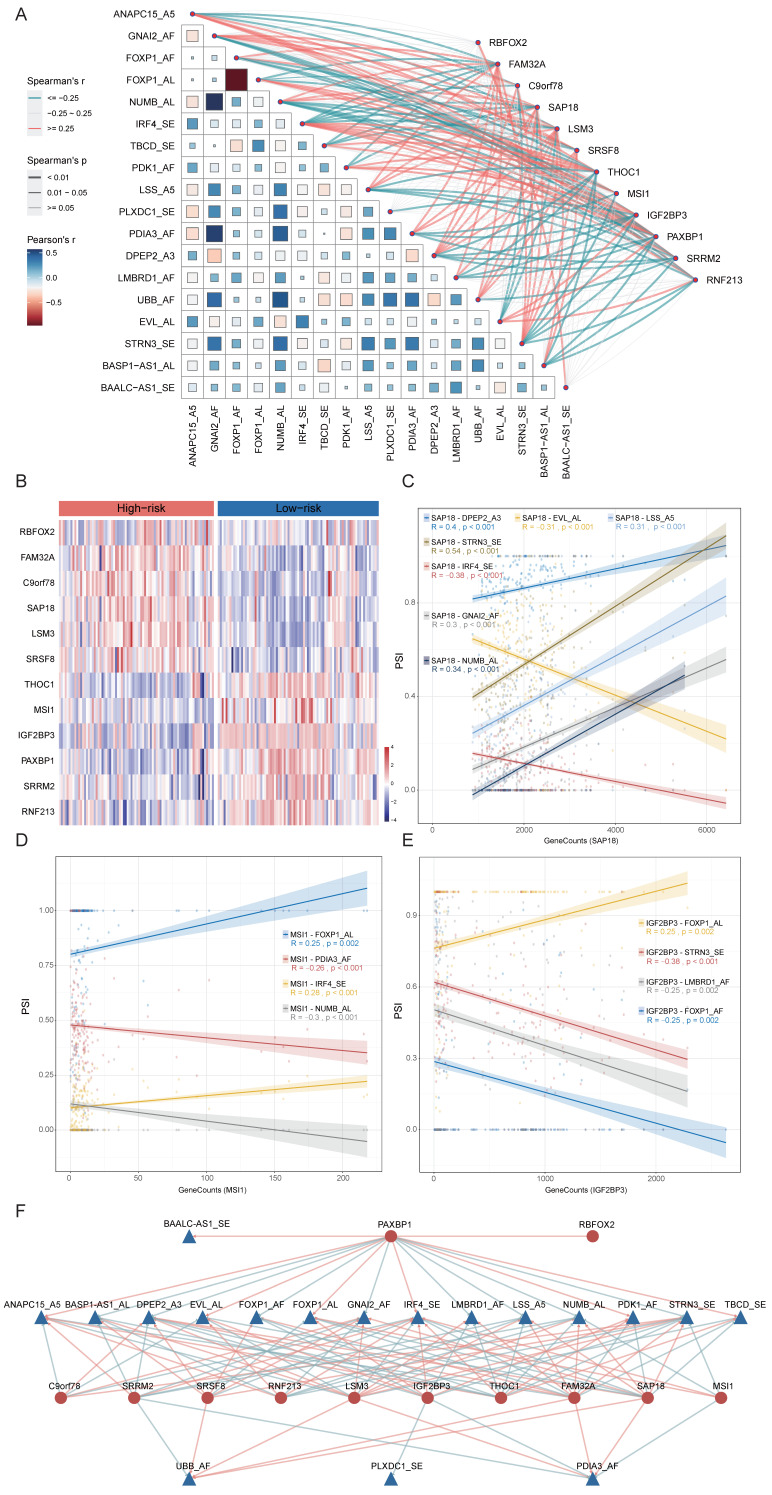
** Construction of the 18-AS and Splicing Factor Regulatory Network and Determination of Candidate Drugs Based on Splicing Factors.** (A) Correlation network between 18-AS and splicing factors. The heatmap's color represents the Pearson correlation coefficient among 18-AS, with darker colors indicating higher correlation. The size of squares in the heatmap signifies the significance of the Pearson correlation among 18-AS, with larger squares indicating greater significance. The width of the network lines represents the Spearman test correlation between SFs and 18-AS, with wider lines indicating stronger correlations. The color of the network lines represents the significance level from the Spearman test. (B) Heatmap of differential expression profiles of splicing factors between 18-AS subgroups. (C-E) Correlation between splicing factors SAP18/MSI1/IGF2BP3 and AS events. (F) Regulatory network between 18-AS and SFs. Triangles (blue) represent AS events, circles (red) represent splicing factors, red lines indicate positive correlation between SFs and AS events, and green lines indicate negative correlations.

The process of alternative splicing is highly organized and regulated by *trans*-acting factors and *cis*-regulatory elements. Splicing factors (SFs), as *trans*-acting factors, influence exon and splice site selections by recognizing *cis*-regulatory elements within pre-mRNA^39^. To explore the potential upstream regulatory network of 18-AS events, we collected splicing factors defined in previous studies^32^ and calculated the correlation between SFs and 18-AS events using correlation analysis. By filtering with an absolute correlation coefficient R ≥ 0.25 and *p* <0.05, we identified a series of SFs related to 18-AS (Fig. 7A). Further analysis of differential expression of SFs between high and low 18-AS patients, combined with correlation analysis, led to the selection of 12 SFs (Fig. 7B), with RBFOX2, LSM3, SRSF8, SAP18, FAM32A, C9orf78 upregulated, and MSI1, IGF2BP3, THOC1, SRRM2, RNF213, PAXBP1 downregulated in high 18-AS patients. We focused on SAP18, MSI1, and IGF2BP3, which have been reported in hematological tumor research (Fig. 7C-E). SAP18, as an integral splicing component of the exon junction complex (EJC)-related apoptosis and splicing associated protein (ASAP)/PNN-RNPS1-SAP18 (PSAP) complex^40^, is associated with seven AS events including IRF4_SE, DPEP2_A3 *etc*. Previous studies reported that SAP18 is involved in the apoptosis signaling pathway in T-cell acute lymphoblastic leukemia (T-ALL)^41^ and non-Hodgkin lymphoma^42^, affecting disease progression. MSI1, involved in maintaining cell stemness and oncogenesis, has been shown to bind to the 3'-untranslated region of NUMB, a Notch inhibitor, thereby regulating the Notch signaling pathway^43^, and is associated with four AS events including IRF4_SE, NUMB_AL in our network. IGF2BP3, involved in the localization, stability, and translational regulation of target RNAs, is related to four AS events including FOXP1_AL, STRN3_SE *etc*., and its low expression in B-ALL has been reported to indicate lower survival rates in pediatric B-ALL^44^, consistent with our findings. In our constructed SF-AS network (Fig. 7F), the relationships between SFs and AS events are not only one-to-one but also many-to-one or many-to-many.

### Validation of Candidate Drugs For High 18-AS Patients

**Figure 8:**
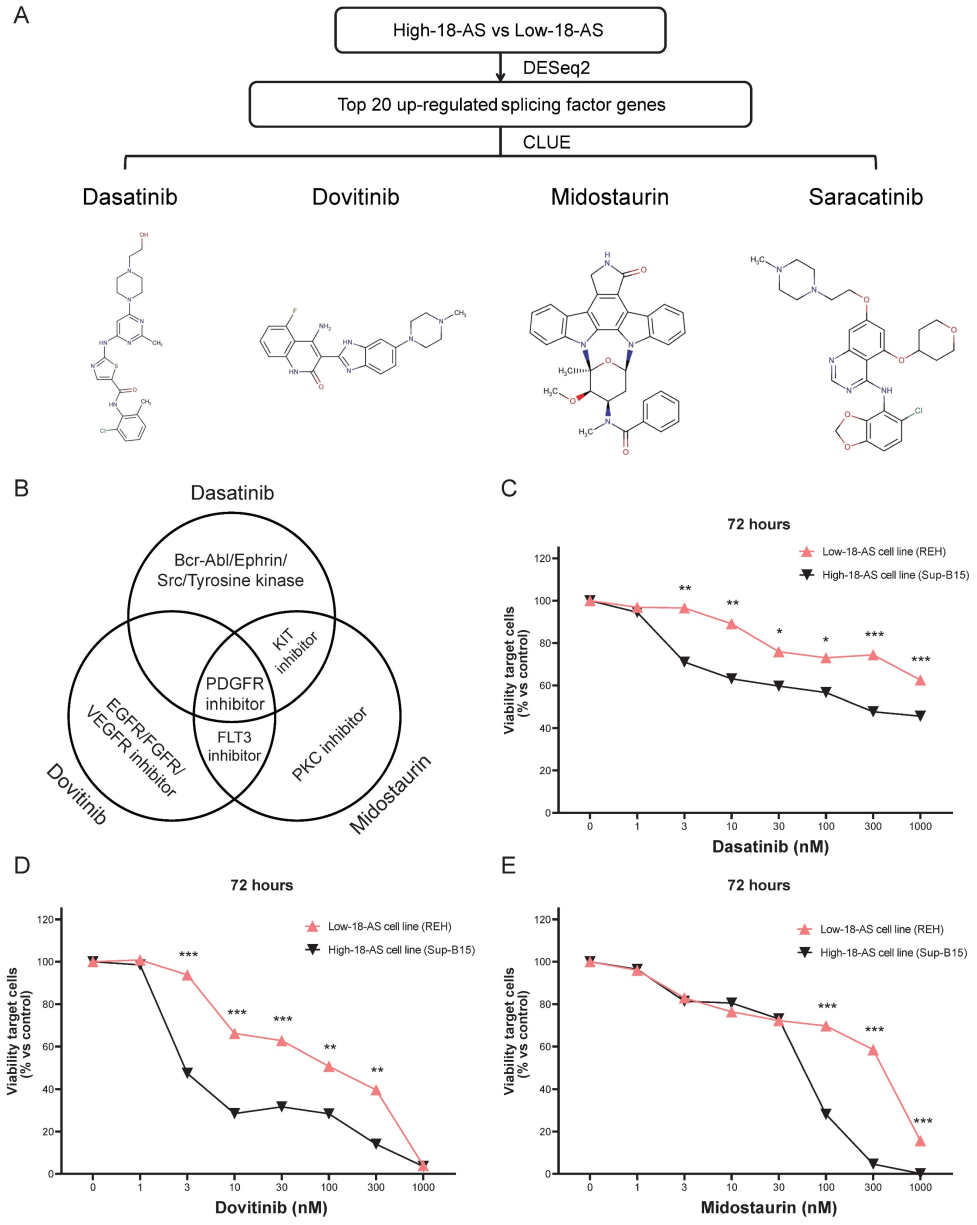
** Validation of Candidate Drugs for High 18-AS Patients.** (A) Workflow for screening potential drugs for B-ALL treatment based on splicing factors, including the chemical structures of candidate drugs and their IC_50_ value distributions across different 18-AS subgroups. (B) Venn diagram showing the mechanisms of action (MOA) for Dasatinib, Dovitinib and Midostaurin. (C-E) Low-18-AS cells line (REH) and High-18-AS cells line (Sup-B15) were treated with three different drugs (Dasatinib, Dovitinib, or Midostaurin) at various concentrations for 72 hours, respectively. Data are presentedas means ± SEM. Statistical analysis was conducted with a two-way ANOVA type test, with significance levels indicated as *, *P* < 0.05; **, *P* < 0.01; ***, *P* < 0.001; n=3.

Given the poor survival rate of high 18-AS patients, we aimed to identify effective drugs that could potentially improve disease progression in these patients. The top 20 upregulated SFs in high 18-AS patients were used as predictive targets, and potential drugs reversing these gene expressions were screened through CLUE analysis. The results highlighted four drugs (Dasatinib, Dovitinib, Midostaurin and Saracatinib) as good candidates in B-ALL treatment (Fig. 8A). Midostaurin and Dasatinib share a common action as KIT inhibitors, while Midostaurin and Dovitinib as FLT3 inhibitors (Fig. 8B). Detailed information about the candidate drugs is listed in Table S2.

To further validate the efficacy of these candidate drugs, we conducted a CCK-8 assay on B-ALL cell lines. The Sup-B15 cell line, which corresponds to the BCR::ABL1 fusion subtype and predominantly represents high-18-AS, and the REH cell line, which corresponds to the ETV6::RUNX1 fusion subtype and predominantly represents low-18-AS, were chosen to ensure that our experimental validation reflects the clinical relevance of the 18-AS stratification (Fig. 4A). The CCK-8 assay results demonstrated significant differences in drug sensitivity between high 18-AS and low 18-AS cell lines. High 18-AS cell lines (Sup-B15) exhibited markedly lower viability compared to low 18-AS cell lines (REH) when treated with Dasatinib, Dovitinib, and Midostaurin at various concentrations. For Dasatinib, the viability of high-18-AS cells decreased significantly at concentrations as low as 1 nM (*p* < 0.01), while low 18-AS cells required higher concentrations to achieve similar effects. Similarly, both Dovitinib and Midostaurin demonstrated a dose-dependent decrease in cell viability, with high 18-AS cells showing greater sensitivity at tested concentrations (*p* < 0.01). All these results were in line with our bioinformatic analysis.

## Discussion

Alternative splicing is a highly regulated and coordinated molecular mechanism and is considered a key characteristic of cancer occurrence, differentiation, and treatment, gradually attracting widespread attention in various diseases, including B-ALL. Researchers have developed prognostic models or molecular typing models based on alternative splicing in cancers such as acute myeloid leukemia (AML)^45^, renal cell carcinoma^46^, lung adenocarcinoma^47^, uterine sarcoma^48^, and pan-gastrointestinal adenocarcinoma^49^. Furthermore, studies analyzing alternative splicing events across 33 types of cancer have shown that alternative splicing events can be used for molecular subtyping of cancers, revealing significant clinical relevance of abnormal alternative splicing events to cancer^50^. However, the applicability of alternative splicing as a prognostic indicator for B-ALL has not yet been reported. To address this gap, our study integrates high-throughput RNA-seq datasets to explore the relationship between AS events and B-ALL prognosis, disease progression, and drug benefits, and we have also established an SF-AS regulatory network to identify effective therapeutic drugs.

We employed SUPPA2 for AS event identification due to its recognized efficacy in event-based analysis. SUPPA2 was chosen based on its demonstrated performance in detecting AS events with high precision and recall. In our previous benchmarking study^51^, SUPPA2 was shown to be among the top-performing tools for event-based splicing analysis. However, we acknowledge the limitations of relying on a single algorithm and the potential for false positives associated with SUPPA2. To mitigate the risk of false positives, we implemented parameter filtering during the execution, ensuring that only AS events with sufficient transcript support were considered. This approach helped to refine the AS event detection and reduce the likelihood of false positives. The robustness of our findings is supported by the comprehensive nature of the dataset and the consistency of the 18-AS signature across multiple independent cohorts. Additionally, we validated eight AS events using RT-PCR in our high 18-AS and low 18-AS cell lines (Fig. S6) and generated Sashimi plots from the RNA-seq data of patients (Fig. S7) to offer a comprehensive validation of our identified AS events.

In this study, we identified prognostic-related AS events using univariate Cox analysis, consensus clustering combined with WGCNA. To efficiently integrate these prognostic-related AS events, we utilized the PSI matrix of AS events, identifying the B-ALL prognostic 18-AS signature through an integration pipeline comprising ten different machine learning algorithms. We fitted 101 machine learning algorithms to the training set, among which models constructed with the CoxBoost and RSF algorithms exhibited the most outstanding performance across three datasets. Integrating a variety of methods based on different machine learning algorithms can combine the strengths of various algorithms, enhancing the predictive performance and generalizability of the model. By integrating algorithms, multiple machine learning algorithms can be combined to fit and predict data together. This integrated approach helps find prognostic markers with consistent performance across Chinese, Japanese and European datasets for B-ALL prognosis. At the same time, reducing the dimensionality of variables through integrated algorithms can simplify the model, making it easier to understand and apply. Furthermore, our ROC and DCA curve analyses indicate that 18-AS maintains accuracy and stable performance across three independent datasets, demonstrating its significant potential for clinical application. These findings further support the broad applicability and independent prognostic value of the 18-AS signature in B-ALL patients. By validating the signature across various subtypes and age groups, we demonstrate that the 18-AS signature is not only a marker associated with known high-risk subtypes but also an independent prognostic tool with significant clinical relevance.

Given the significant prognostic differences between the 18-AS subgroups, we subsequently explored the potential mechanisms. Within 18-AS, we identified key genes involved in B-cell growth and development such as FOXP1^52^, ^53^, IRF4^54^, ^55^, PDK1^56^, ^57^ incorporated into these prognostic signatures. Pathway enrichment results indicated that genes undergoing AS participated in regulating various pathways including immune cell activation and B-cell apoptosis. Previous studies have shown that the activity of multiple protein isoforms produced by alternative FOXP1 promoters might regulate B-cell maturation, and FOXP1 alternative splicing has been proven to affect the progression of diffuse large B-cell lymphoma^58^. IRF4, a key factor in regulating B-cell development, can negatively regulate pre-B cell proliferation and promote immunoglobulin locus rearrangement^59^, and studies have indicated that IRF4 deficiency accelerates the progression of BCR::ABL positive B-ALL in mice^60^. Additionally, our immune infiltration analysis revealed that patients with a low 18-AS score demonstrated higher immune infiltration abundance. Concurrently, both adult and pediatric high 18-AS patients exhibited defects in V(D)J rearrangement, suggesting they might face more severe B-cell differentiation disorders, leading to impaired B-cell function. More importantly, our drug sensitivity analysis revealed that high 18-AS patients had higher IC_50_ values for six commonly used B-ALL drugs including Nelarabine, Vincristine and Imatinib, which is supportive of our AS-based prediction.

Studies on pan-cancer AS events have discovered many differences in selective splicing compared to normal cells, which are characteristic of individual cancer types and can be used to design immune therapy interventions^61^. Therefore, we extended the TIDE algorithm to B-ALL patients to assess the differences in immune therapy responses between the 18-AS subgroups, with results still showing significantly lower immune therapy benefits in the high 18-AS group compared to the low 18-AS group. Given the recent rapid development of CAR T-cell therapy in B-ALL^62^, it will be of great interest to consider whether a similar AS-based risk assessment could be implemented prior to the use of immune therapy. To explore more effective treatment strategies for high 18-AS patients, we also characterized the potential regulatory splicing factors and developed an SF-AS regulatory network, providing some references for exploring the upstream mechanisms of 18-AS. Splicing factors can serve as predictive targets for drug screening, as demonstrated by Wan LD et al.'s study, where SRSF6, an SR protein overexpressed in colorectal cancer, promotes tumor progression by regulating AS. Virtual drug screening identified the SRSF6-targeting inhibitor indacaterol, which was evaluated *in vitro* and *in vivo* for its antitumor effects, showing that indacaterol could serve as a novel therapeutic agent by targeting SRSF6 to regulate AS and thereby inhibit CRC progression^63^. Based on this, we screened for drugs targeting the splicing factors highly expressed in high 18-AS patients in B-ALL cell lines, identifying four highly confident candidate drugs. Through access to the database^64^, we found that these compounds target specific pathways and genes crucial for regulating various cellular processes (Fig. S8). For example, Dasatinib primarily influences signal transduction, the apoptotic process, and leukocyte activation. It targets genes such as ABL1, ABL2, BCR and SRC. These genes interact with splicing factors like SAP18, FAM32A, and RBFOX2, playing significant roles in nucleic acid metabolic processes and cell-cell adhesion regulation. Dovitinib affects pathways involved in cellular metabolic processes, cell population proliferation, and the vascular endothelial growth factor signaling pathway. Its target genes interact with splicing factors such as C9orf78, IGF2BP3, and SAP18, crucially influencing cellular metabolism and proliferation. Midostaurin impacts the regulation of the MAPK cascade, cell population proliferation, and the vascular endothelial growth factor signaling pathway. It targets key genes like FLT1, FLT3, KIT, PDGFRB, and VEGFA, which interact with splicing factors including LSM3, SAP18, and THOC1. These interactions are pivotal in regulating cell proliferation and metabolic processes. Saracatinib regulates apoptosis and cell adhesion by targeting ABL1 and LCK, interacting with FAM32A. To assess the compounds' clinical potential, we performed CCK-8 assays. The results showed significant differences in drug sensitivity between high 18-AS and low 18-AS cell lines, confirming our findings' clinical relevance. Dasatinib is currently approved for use in chronic myelogenous leukemia (CML) and ALL, Midostaurin for AML and mast cell leukemia, while Dovitinib is not yet included in leukemia treatment guidelines. Studies have proven that Midostaurin, as a KIT inhibitor, can significantly prolong progression-free survival in ALL patients when used in combination^65^. Concurrently, Dovitinib, targeting FLT1, EGFR, FLT3 and KIT, acts as a EGFR inhibitor and FLT3 inhibitor, with previous research showing Dovitinib showed treatment efficacy in naïve and imatinib-resistant BCR::ABL(+) leukemia cells^66^. However, it is worthy to point out that the practical applicability of these drugs in B-ALL patients still requires vigorous exploration in future studies through experiments.

In summary, this study developed a robust set of 18-AS signatures using bioinformatics and machine learning algorithms, providing valuable insights for assessing the immune microenvironment and clinical outcomes in B-ALL patients. These findings open new avenues for personalized treatment strategies in B-ALL. However, our study has limitations, including the need for a broader cohort of B-ALL patients to confirm the 18-AS prognostic value and further exploration of the splicing regulatory mechanisms and target drugs. Despite these limitations, our research enhances the understanding of AS events in B-ALL, identifies key prognostic AS events, and highlights potential therapeutic drugs.

## Supplementary Material

Supplementary methods, figures and tables.
