# Supplementary material for: Integrative alternative splicing analysis reveals new prognosis signature in B-cell acute lymphoblastic leukemia
Source: Int J Biol Sci. 2024 Aug 19;20(11):4496–512. doi: 10.7150/ijbs.98899 (PMC11380455; doi:10.7150/ijbs.98899)
Supplement: Supplementary file 1 — Supplementary methods, figures and tables. [file ijbsv20p4496s1.pdf]

SUPPLEMENTAL METHODS, FIGURES , LEGENDS AND TABLES

Supplemental Methods

Data preprocessing and expression quantification

The preprocessing procedures involved quality assessment by FastQC (v 0.11.9) and MultiQC (v 1.10), as well as reads trimming by trim_galore (v 0.6.7). Before trimming, quality of each raw file was first assessed by FastQC and summarized by MultiQC. Then, adapters and low-quality ends (quality score <25 and reads length <36) were removed by trim_galore, with parameters '*-q 25 --phred33 --length 36 --stringency 3 -paired*'. The trimmed reads were reanalyzed by FastQC to confirm their compliance with quality requirements. Following the preprocessing procedures, salmon (v 0.13.1) was employed to quantify the abundance of transcript isoforms with genome annotation file (GRCh38.109.gtf) with parameters '*salmon quant -i GRCh38.109.gtf -l A --gcBias --seqBias -1 ${id}_R1.fq.gz -2 ${id}_R2.fq.gz --dumpEq --numBootstraps 100 --validateMappings*'.

Detection of alternative splicing events

AS analysis was performed by SUPPA2 (v 2.3) based on transcript expression levels, with parameters '*suppa.py psiPerEvent -GRCh38.109.all.events.ioe -e ./salmon_output/iso_tpm.txt -f 5 -o AS*'. The '-f' parameter sets a minimum total expression threshold for the transcripts involved in the event, filtering out events that do not reach this expression value. This ensures that only AS events with sufficient transcript support are considered, thereby reducing the likelihood of false positives. The percent spliced in (PSI) values, ranging from 0 to 1, were used to quantify AS events. PSI was calculated by dividing the read counts of long isoforms by the sum of both short and long isoforms.

Immunoglobulin Repertoire V(D)J Rearrangement Analysis

MiXCR (v4.0) was used to extract immunoglobulin repertoire information, with parameters *'mixcr analyze rnaseq-cdr3 --species hsa ${id}_R1.fq.gz ${id}_R2.fq.gz mixcr_output/${id}*'.

The Convert program of VDJtools (v 1.2.1) was used for format conversion, with parameters '*java -jar vdjtools-1.2.1.jar Convert -S mixcr ${id}.clones_IGH.tsv VDJtoolsInput*'. The PlotFancyVJUsage and RarefactionPlot programs were utilized for visualization, with parameters'*java -jar vdjtools-1.2.1.jar PlotFancyVJUsage VDJtoolsInput VDJtoolsOutput/${id}*' and '*java -jar vdjtools-1.2.1.jar RarefactionPlot -m metadata.txt VDJtoolsOutput/IGH*'.

Supplemental Figure 1

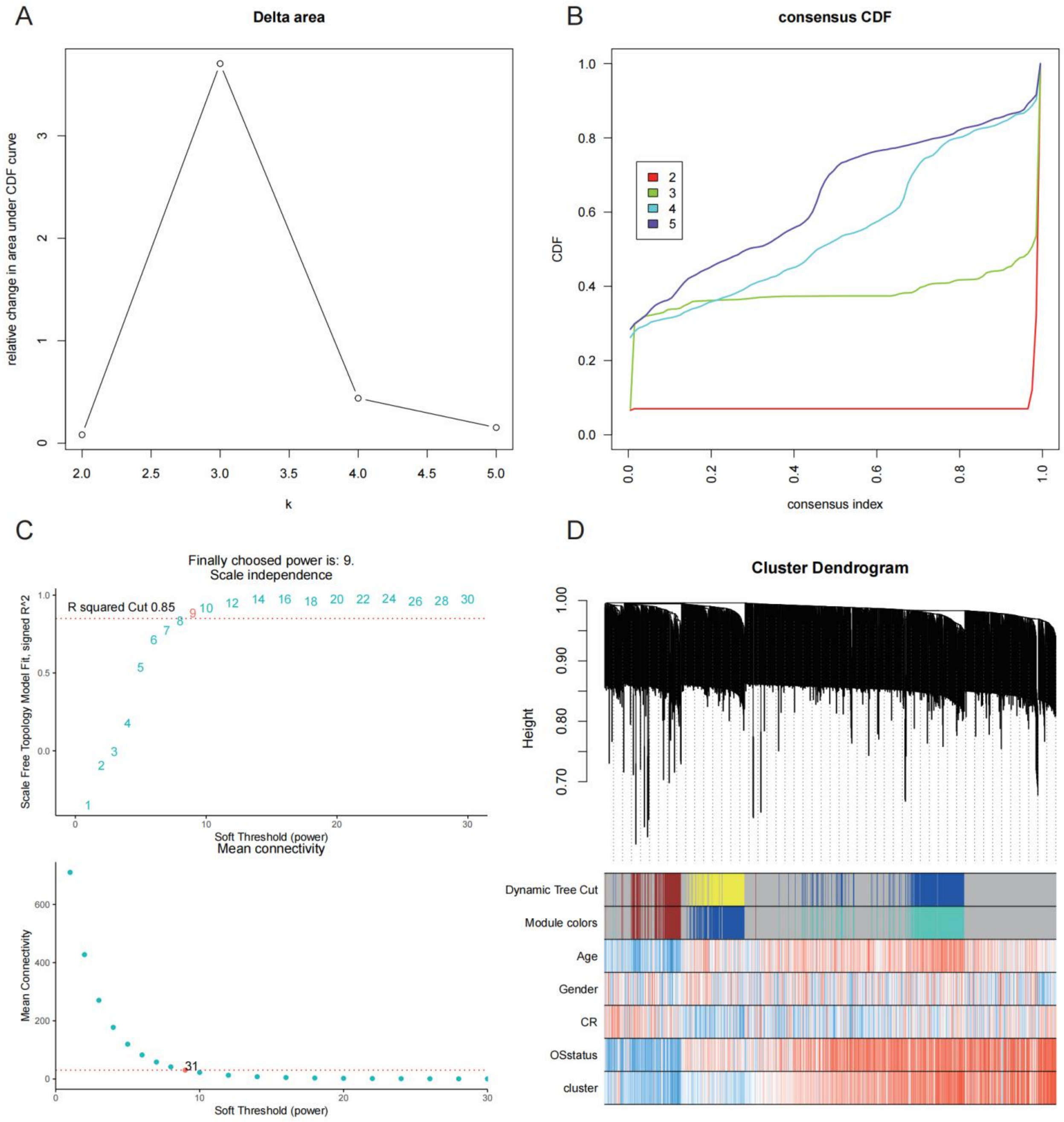

Figure S1: Identification of the optimal k value and soft thresholding.

(A) Relative change in the area under the CDF curve for k = 2-4. (B) CDF curves of the consensus matrix for k = 2-4, depicted in varying colors to represent different cluster numbers (k). (C) Identification of the optimal soft threshold to achieve a scale-free topology structure. The upper panel shows that the minimal soft threshold value of 9 corresponds to a scale-free fit index of 0.85. The lower panel illustrates network connectivity across different soft threshold values. (D) Clustering dendrogram of alternative splicing events constructed using the optimal soft threshold, depicting the co-expression network that clusters AS events into modules based on their expression patterns.

Supplemental Figure 2

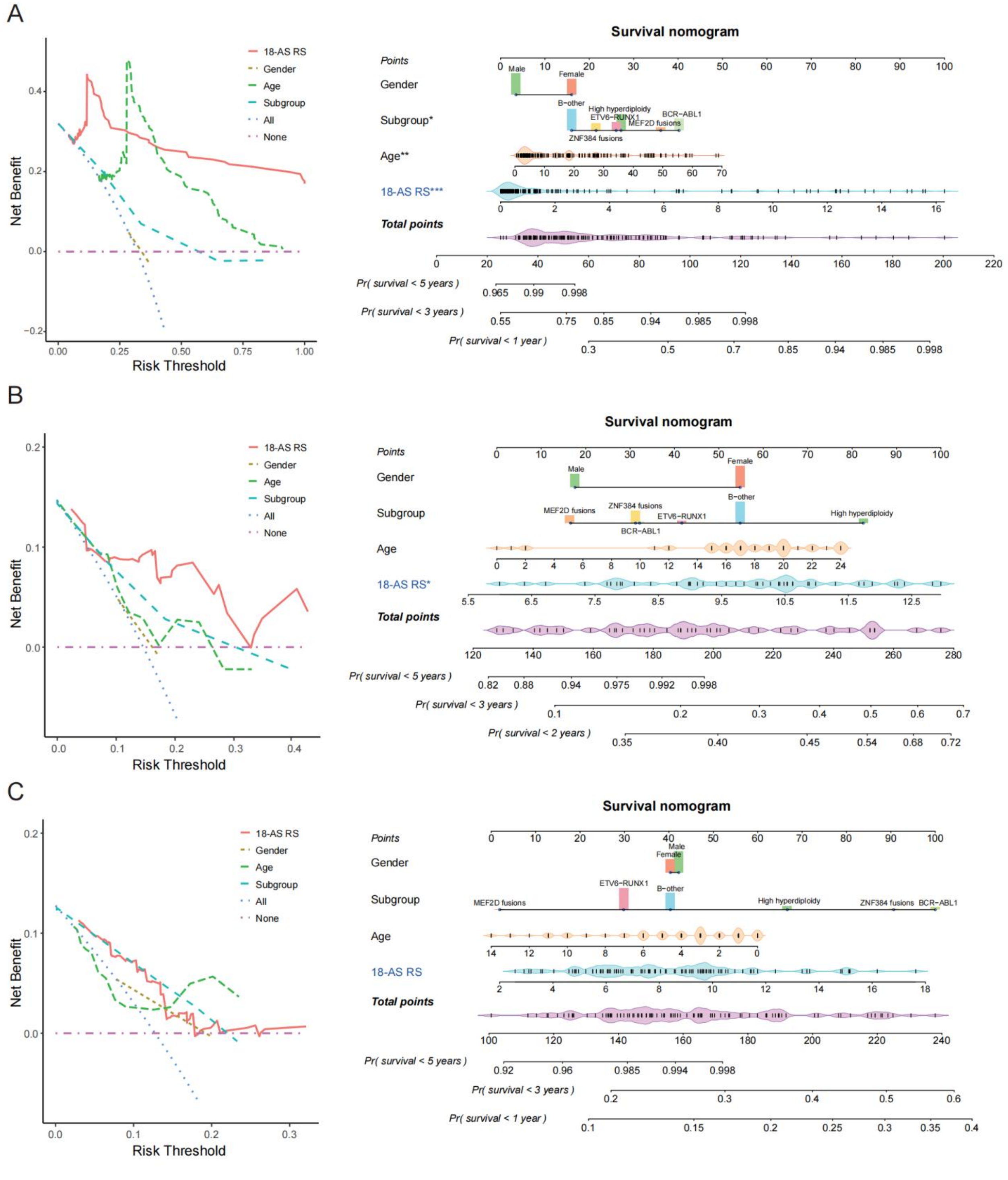

Figure S2: Decision curve analysis (DCA) and nomograms across B-ALL Cohorts.

(A-C) Decision curve analysis (DCA) for the CGA (A), JGA (B), and EGA (C) cohorts are presented on the left, demonstrating that the 18-AS signature offers greater net survival benefits compared to other parameters. On the right, nomograms for each dataset are depicted, providing a visual representation of the prognostic model that quantifies the survival probability based on the 18-AS signature and other clinical features.

Supplemental Figure 3

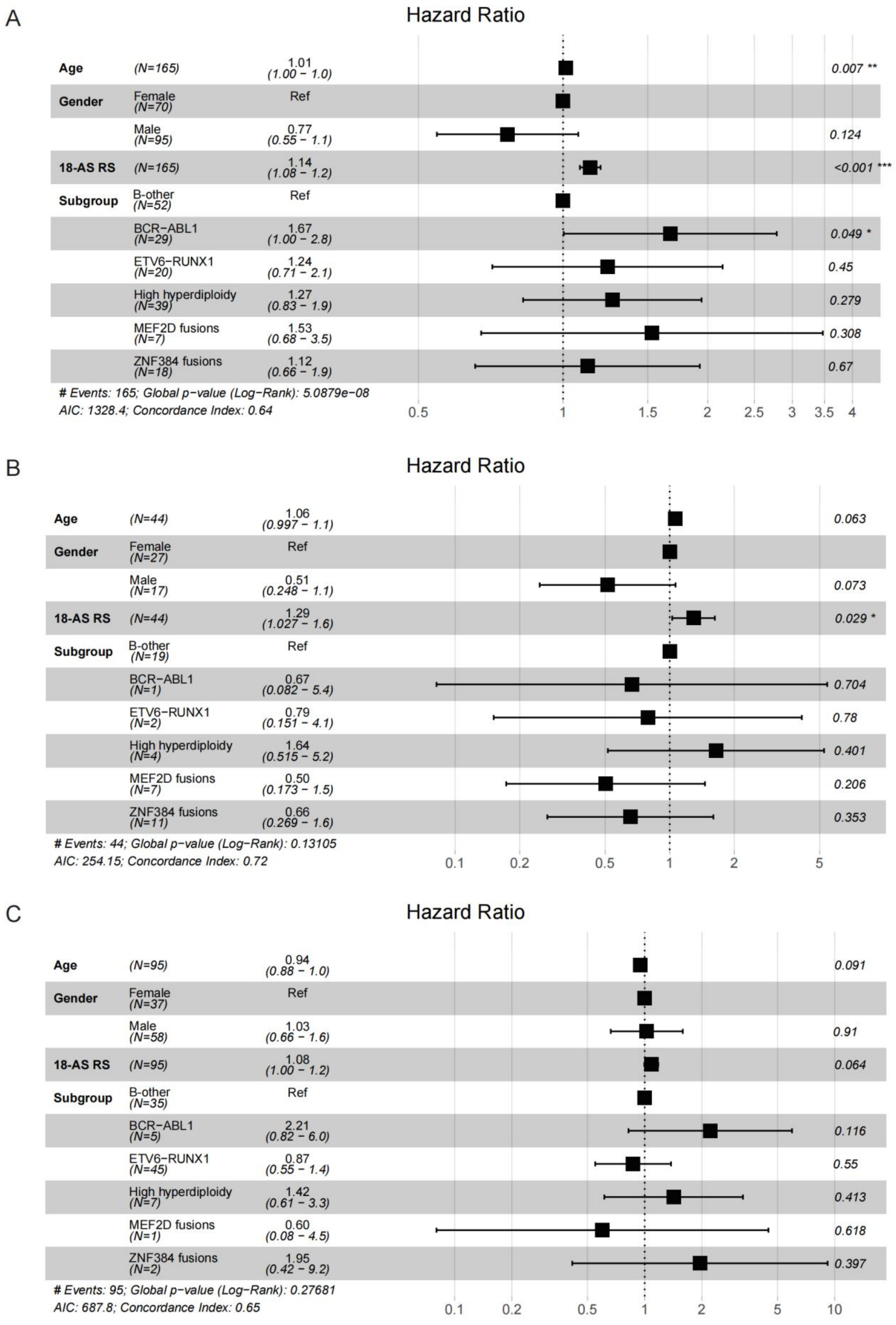

Figure S3: Forest plots across B-ALL cohorts.

(A-C) Forest plots for the CGA (A), JGA (B), and EGA (C) cohorts, illustrating the hazard ratios (HRs) and confidence intervals for the prognostic impact of the 18-AS signature and other clinical variables on patient outcomes.

Supplemental Figure 4

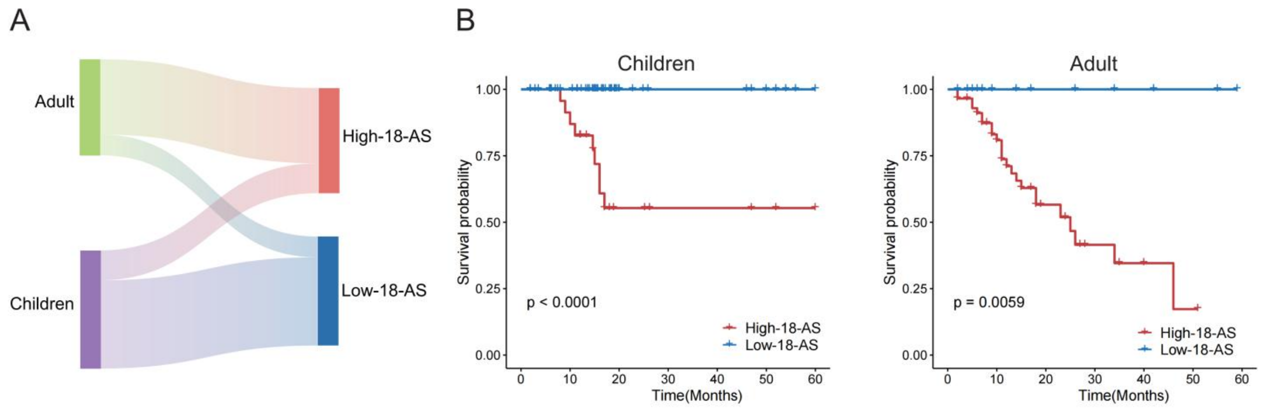

Figure S4: Prognostic Value of the 18-AS in Pediatric and Adult B-ALL Patients.

(A) Mulberry chart of patient age distribution based on 18-AS, where the width of the bars is proportional to the quantity ratio. (B) Kaplan-Meier survival curves for patients with the different age stratified by high-18-AS and low-18-AS groups.

Supplemental Figure 5

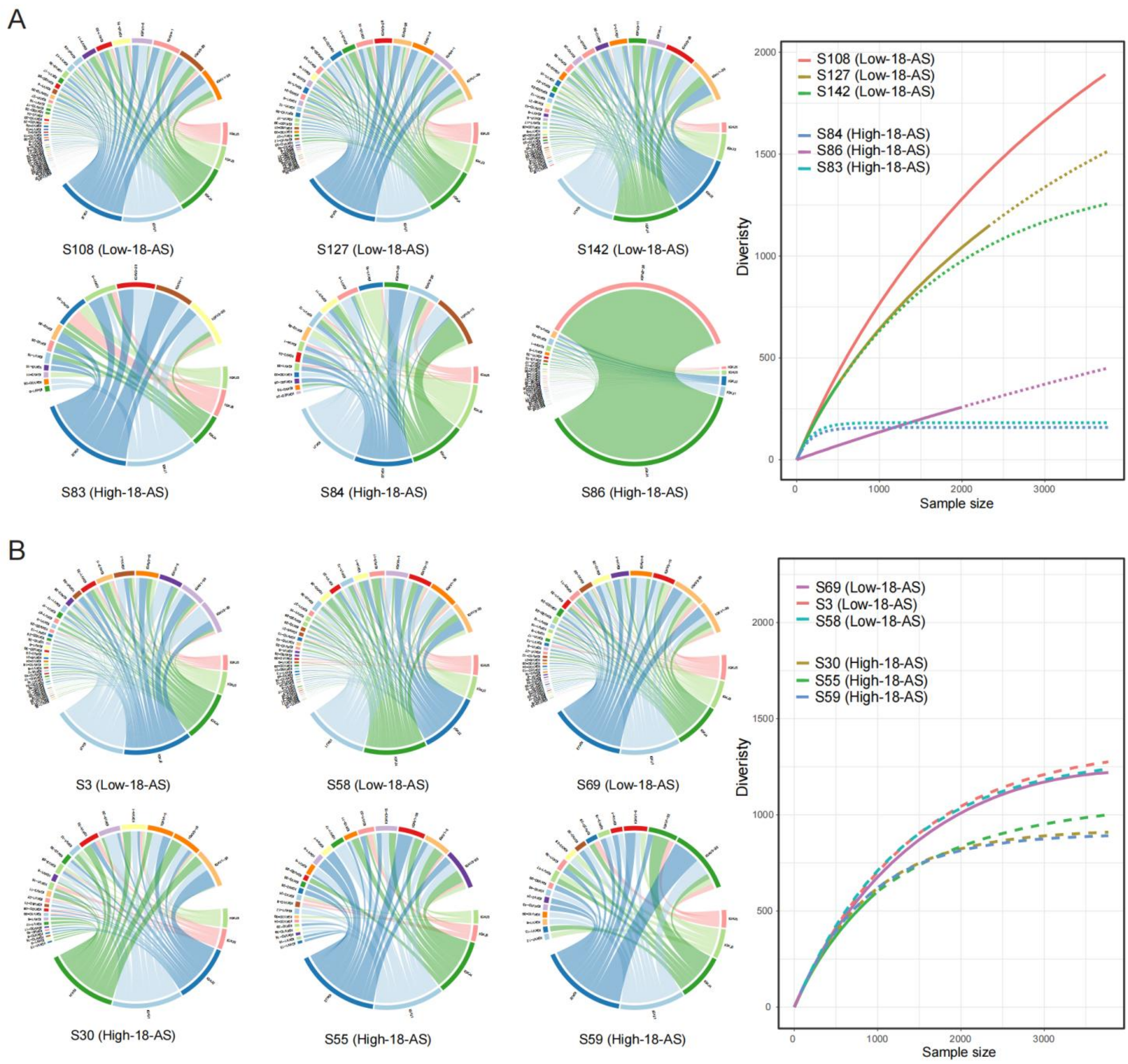

Figure S5: Analysis of V_kJ_k rearrangements in B-ALL patients.

(A) Circos plot of V_kJ_k rearrangement in pediatric patients across different 18-AS subgroups, where the width of the bands correlates with the frequency of rearrangement events. The diversity of V_kJ_k rearrangements in patients with different 18-AS subgroups is summarized in the curve plot on the right. (B) Circos plot of V_kJ_k rearrangement in adult patients across different 18-AS subgroups, similarly illustrating the proportional relationship between band width and rearrangement event frequency. The diversity of V_kJ_k rearrangements in patients with different 18-AS subgroups is summarized in the curve plot on the right.

Supplemental Figure 6

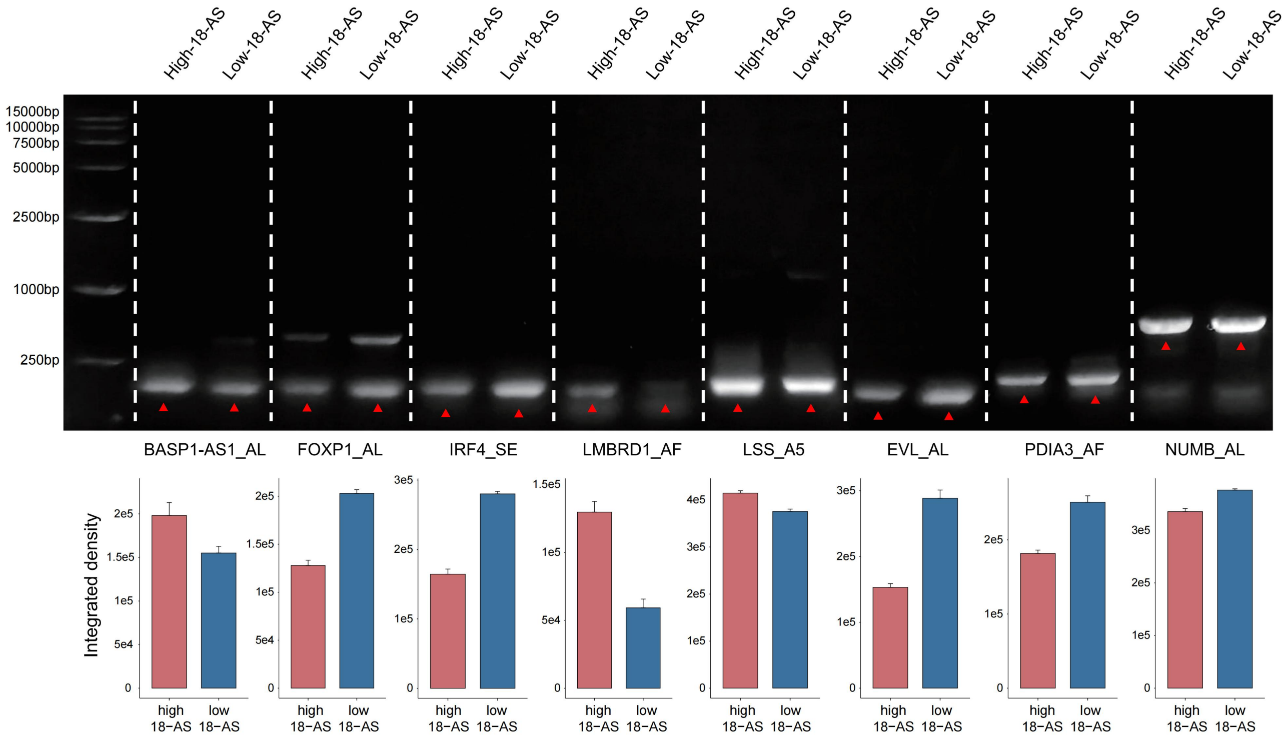

Figure S6: RT-PCR validation of AS events in high-18-AS and low-18-AS cell lines.

Top panel, gel electrophoresis results of RT-PCR for eight validated AS events in Sup-B15 (high-18-AS) and REH (low-18-AS) cell lines. Bottom panel, the quantification of integrated density values corresponding to the bands in the top panel.

Supplemental Figure 7

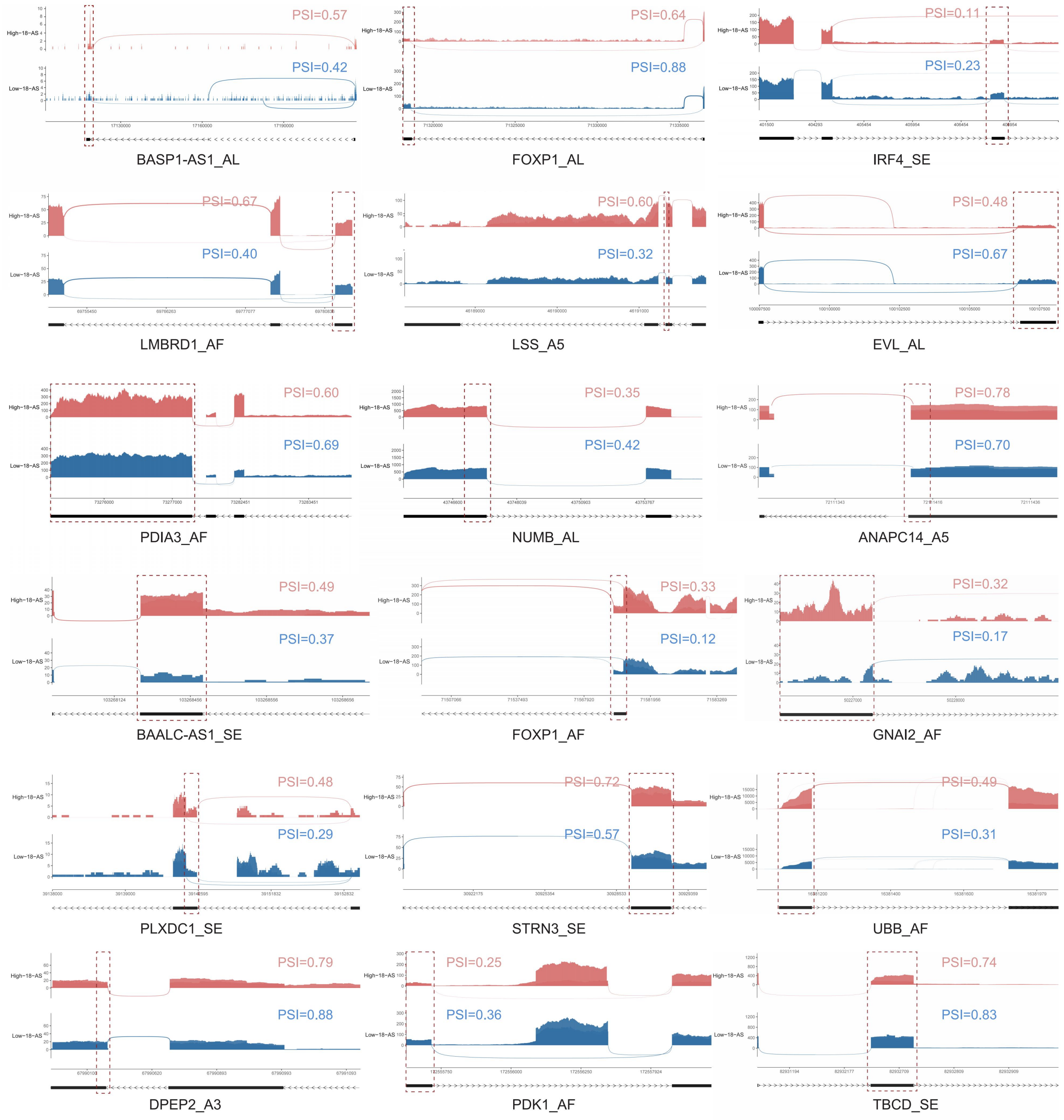

Figure S7: Sashimi plots of 18-AS in patients.

Read coverage and junctions are shown the splicing patterns with PSI values to indicate the exon inclusion levels.

Supplemental Figure 8

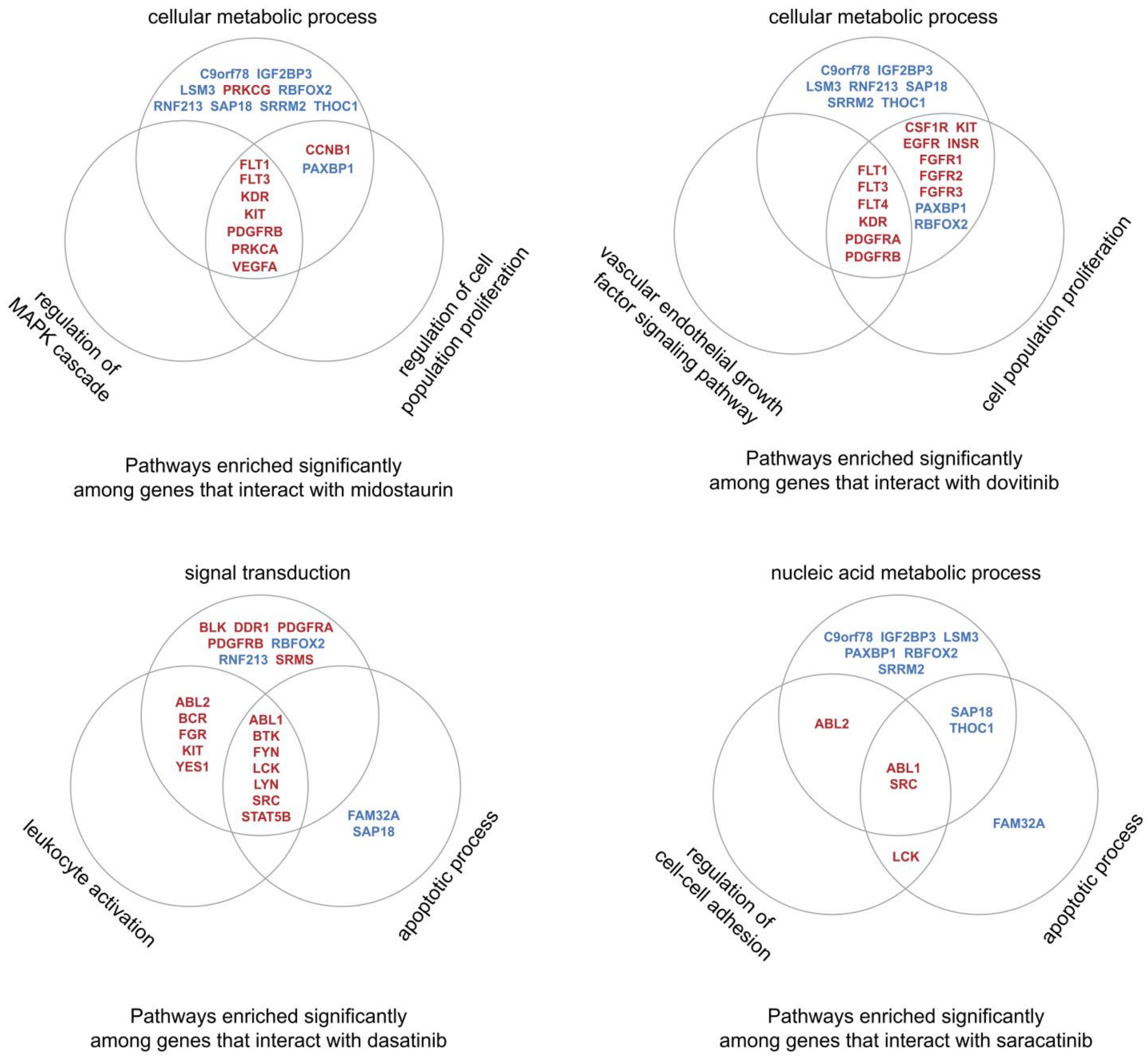

Figure S8: Pathway enrichment and interacting genes analysis of the candidate compounds.

Target genes of the compounds are shown in red, with splicing factor in blue.

Table S1.Basic information of the CGA cohort

Sample	RNA-Seq.Library	Age	Gender	Data.Warehouse	CR(0=no, 1=yes)	OSmonths	OS.status	Consensus Cluster	18-AS-group
S1	mRNA	27.54	Male	CGA	0	40	0	C1	High-18-AS
S10	mRNA	47.03	Female	CGA	1	55	0	C1	Low-18-AS
S100	mRNA	5.81	Male	CGA	1	60	0	C1	Low-18-AS
S101	mRNA	6.43	Female	CGA	1	60	0	C1	Low-18-AS
S102	mRNA	7.62	Female	CGA	1	60	0	C1	Low-18-AS
S103	mRNA	2.91	Female	CGA	1	60	0	C1	Low-18-AS
S104	mRNA	8.88	Female	CGA	1	60	0	C1	Low-18-AS
S105	mRNA	3.92	Male	CGA	1	60	0	C1	Low-18-AS
S106	mRNA	12.98	Female	CGA	1	60	0	C1	Low-18-AS
S107	mRNA	11.4	Male	CGA	1	60	0	C1	High-18-AS
S108	mRNA	3.2	Male	CGA	1	56	0	C1	Low-18-AS
S109	mRNA	11.1	Male	CGA	1	52	0	C1	High-18-AS
S11	mRNA	21.91	Male	CGA	1	46	1	C1	High-18-AS
S110	mRNA	1.02	Female	CGA	1	60	0	C1	Low-18-AS
S111	mRNA	2.39	Male	CGA	1	60	0	C1	Low-18-AS
S112	mRNA	2.74	Male	CGA	1	60	0	C1	Low-18-AS
S113	mRNA	12.93	Male	CGA	1	60	0	C1	High-18-AS
S114	mRNA	5.15	Male	CGA	1	60	0	C1	Low-18-AS
S115	mRNA	13.83	Female	CGA	1	60	0	C1	Low-18-AS
S116	mRNA	6.08	Male	CGA	1	60	0	C1	Low-18-AS
S117	mRNA	4.35	Female	CGA	1	50	0	C1	Low-18-AS
S118	mRNA	5.63	Male	CGA	1	46	0	C1	Low-18-AS
S119	mRNA	3.71	Male	CGA	1	60	0	C1	High-18-AS
S12	mRNA	32.46	Female	CGA	1	26	1	C1	High-18-AS
S120	mRNA	13.78	Female	CGA	1	47	0	C1	High-18-AS
S121	mRNA	10.59	Male	CGA	1	52	0	C1	Low-18-AS

S122	mRNA	2.88	Female	CGA	1	54	0	C1	Low-18-AS
S123	mRNA	8.87	Male	CGA	1	47	0	C1	Low-18-AS
S124	mRNA	14.92	Female	CGA	1	18	0	C1	Low-18-AS
S125	mRNA	8.78	Male	CGA	1	8	0	C1	Low-18-AS
S126	mRNA	2.1	Female	CGA	1	10.5	0	C1	Low-18-AS
S127	mRNA	2.16	Female	CGA	1	6.2	0	C1	Low-18-AS
S128	mRNA	4.14	Male	CGA	1	7.5	0	C1	Low-18-AS
S129	mRNA	2.91	Male	CGA	1	12.13333333	0	C1	High-18-AS
S13	mRNA	36.26	Male	CGA	1	7	1	C2	High-18-AS
S130	mRNA	13.59	Male	CGA	0	16.7	0	C1	Low-18-AS
S132	mRNA	5.98	Female	CGA	1	11.5	0	C1	Low-18-AS
S133	mRNA	1.92	Female	CGA	1	16.8	0	C1	Low-18-AS
S134	mRNA	4.25	Male	CGA	1	15.6	0	C1	Low-18-AS
S135	mRNA	3.37	Male	CGA	1	19.23333333	0	C1	Low-18-AS
S136	mRNA	4.81	Female	CGA	1	5.833333333	0	C1	Low-18-AS
S137	mRNA	4.07	Male	CGA	1	14.96666667	0	C1	Low-18-AS
S138	mRNA	3.04	Male	CGA	1	15.1	0	C1	Low-18-AS
S139	mRNA	1.09	Male	CGA	1	14.63333333	1	C1	High-18-AS
S14	mRNA	44.32	Male	CGA	1	35	0	C1	High-18-AS
S140	mRNA	5.07	Male	CGA	1	18.26666667	0	C1	Low-18-AS
S141	mRNA	3.65	Female	CGA	1	13.23333333	0	C1	Low-18-AS
S142	mRNA	2.42	Male	CGA	1	13.63333333	0	C1	Low-18-AS
S143	mRNA	9.93	Male	CGA	1	14.66666667	0	C1	High-18-AS
S145	mRNA	9.47	Male	CGA	1	19.16666667	0	C1	Low-18-AS
S146	mRNA	13.81	Male	CGA	1	19.5	0	C1	Low-18-AS
S147	mRNA	3.96	Female	CGA	1	13.96666667	0	C1	Low-18-AS
S148	mRNA	6.98	Female	CGA	1	11.4	0	C1	Low-18-AS
S149	mRNA	1.18	Male	CGA	1	13.36666667	0	C1	High-18-AS
S150	mRNA	7.75	Female	CGA	1	16.4	0	C1	Low-18-AS

S151	mRNA	2.23	Female	CGA	1	15.1	0	C1	Low-18-AS
S152	mRNA	8.12	Female	CGA	1	15.66666667	0	C1	Low-18-AS
S153	mRNA	13.59	Male	CGA	1	17.2	0	C1	Low-18-AS
S154	mRNA	1.76	Male	CGA	1	19.86666667	0	C1	Low-18-AS
S155	mRNA	4.62	Male	CGA	1	14.53333333	0	C1	Low-18-AS
S156	mRNA	2.16	Male	CGA	1	10.46666667	0	C1	Low-18-AS
S157	mRNA	4.46	Male	CGA	1	14.7	0	C1	Low-18-AS
S158	mRNA	1.73	Male	CGA	1	18.46666667	0	C1	Low-18-AS
S159	mRNA	7.53	Female	CGA	1	12.3	0	C1	Low-18-AS
S16	mRNA	49.23	Female	CGA	1	6	1	C1	High-18-AS
S160	mRNA	4.17	Male	CGA	1	13.6	0	C1	Low-18-AS
S161	mRNA	0.55	Female	CGA	1	20.03333333	0	C1	Low-18-AS
S162	mRNA	3.5	Male	CGA	1	19.03333333	0	C1	Low-18-AS
S163	mRNA	8.63	Female	CGA	1	18.4	0	C1	Low-18-AS
S164	mRNA	11.09	Male	CGA	1	15.36666667	0	C1	Low-18-AS
S165	mRNA	3.32	Female	CGA	1	18.46666667	0	C1	Low-18-AS
S166	mRNA	5.03	Male	CGA	1	18.9	0	C1	High-18-AS
S167	mRNA	1.17	Male	CGA	1	3.533333333	0	C1	Low-18-AS
S168	mRNA	4.88	Female	CGA	1	1.9	0	C1	Low-18-AS
S169	mRNA	3.03	Female	CGA	1	1.966666667	0	C1	Low-18-AS
S17	mRNA	26.74	Male	CGA	1	15	1	C2	High-18-AS
S170	mRNA	2.95	Male	CGA	1	2.866666667	0	C1	Low-18-AS
S171	mRNA	4.19	Male	CGA	1	26.23333333	0	C1	High-18-AS
S172	mRNA	6.42	Female	CGA	1	25.9	0	C1	Low-18-AS
S19	mRNA	37.28	Male	CGA	1	10	1	C2	High-18-AS
S2	mRNA	68.88	Male	CGA	0	25	1	C1	High-18-AS
S20	mRNA	19.33	Female	CGA	1	9	1	C2	High-18-AS
S22	mRNA	31.35	Male	CGA	1	2	0	C1	Low-18-AS
S23	mRNA	18.16	Male	CGA	1	34	0	C1	Low-18-AS

S24	mRNA	51	Female	CGA	1	2	0	C1	High-18-AS
S25	mRNA	23.64	Male	CGA	1	14	1	C2	High-18-AS
S26	mRNA	18.55	Male	CGA	1	26	0	C1	Low-18-AS
S27	mRNA	23.67	Male	CGA	1	28	0	C1	High-18-AS
S28	mRNA	22.79	Female	CGA	1	23	1	C1	High-18-AS
S29	mRNA	27.83	Female	CGA	1	18	0	C1	High-18-AS
S3	mRNA	31.16	Male	CGA	1	59	0	C1	Low-18-AS
S30	mRNA	18.28	Female	CGA	1	12	1	C2	High-18-AS
S31	mRNA	34.23	Female	CGA	1	24	0	C1	High-18-AS
S32	mRNA	59.14	Female	CGA	0	2	1	C2	High-18-AS
S33	mRNA	53.41	Female	CGA	1	19	0	C2	High-18-AS
S34	mRNA	30.42	Female	CGA	1	19	0	C1	High-18-AS
S36	mRNA	68.04	Female	CGA	0	2	1	C2	High-18-AS
S37	mRNA	38.45	Female	CGA	1	11	1	C1	High-18-AS
S38	mRNA	46.75	Female	CGA	1	17	0	C2	High-18-AS
S39	mRNA	18.24	Male	CGA	1	19	0	C1	High-18-AS
S4	mRNA	49.52	Male	CGA	1	18	1	C2	High-18-AS
S40	mRNA	18.16	Female	CGA	1	19	0	C1	High-18-AS
S41	mRNA	27.33	Male	CGA	1	18	0	C1	High-18-AS
S42	mRNA	26.39	Male	CGA	1	17	0	C2	High-18-AS
S43	mRNA	46.8	Female	CGA	1	42	0	C1	Low-18-AS
S44	mRNA	47.04	Male	CGA	1	7	1	C2	High-18-AS
S45	mRNA	42.44	Female	CGA	1	5	1	C2	High-18-AS
S46	mRNA	25.34	Male	CGA	1	35	0	C1	High-18-AS
S47	mRNA	29.2	Male	CGA	1	6	0	C1	High-18-AS
S48	mRNA	31.28	Female	CGA	1	6	0	C2	High-18-AS
S49	mRNA	55.42	Male	CGA	1	10	0	C2	High-18-AS
S5	mRNA	45.18	Female	CGA	1	10	0	C2	High-18-AS
S50	mRNA	51.05	Male	CGA	1	12	0	C1	High-18-AS

S51	mRNA	21.89	Male	CGA	1	17	0	C1	Low-18-AS
S52	mRNA	26.96	Male	CGA	1	11	0	C2	High-18-AS
S53	mRNA	45.29	Male	CGA	1	8	0	C2	High-18-AS
S54	mRNA	17.81	Female	CGA	1	17	0	C1	High-18-AS
S55	mRNA	19.91	Female	CGA	1	11	1	C2	High-18-AS
S56	mRNA	18.44	Female	CGA	1	11	1	C1	High-18-AS
S57	mRNA	18.75	Male	CGA	1	27	0	C1	High-18-AS
S58	mRNA	18.63	Male	CGA	1	2	0	C1	Low-18-AS
S59	mRNA	22.71	Male	CGA	1	5	1	C2	High-18-AS
S6	mRNA	44.77	Female	CGA	1	4	0	C1	Low-18-AS
S60	mRNA	28	Male	CGA	1	5	0	C1	Low-18-AS
S61	mRNA	58.07	Female	CGA	1	15	0	C1	High-18-AS
S62	mRNA	59.87	Male	CGA	0	18	1	C2	High-18-AS
S63	mRNA	36.92	Female	CGA	1	4	0	C1	High-18-AS
S64	mRNA	46.23	Male	CGA	1	7	0	C1	High-18-AS
S65	mRNA	17.49	Female	CGA	1	12	0	C2	High-18-AS
S66	mRNA	36.66	Female	CGA	1	10	0	C1	High-18-AS
S67	mRNA	18.11	Male	CGA	1	11	0	C2	High-18-AS
S68	mRNA	18.24	Male	CGA	1	11	0	C1	High-18-AS
S69	mRNA	57.67	Male	CGA	1	14	0	C1	Low-18-AS
S7	mRNA	26.54	Male	CGA	1	13	1	C2	High-18-AS
S70	mRNA	18.37	Male	CGA	1	9	0	C1	Low-18-AS
S71	mRNA	24.79	Female	CGA	1	2	0	C2	High-18-AS
S72	mRNA	27.46	Male	CGA	1	9	0	C2	High-18-AS
S73	mRNA	28.31	Female	CGA	1	8	0	C2	High-18-AS
S74	mRNA	29.98	Female	CGA	1	7	0	C1	High-18-AS
S75	mRNA	43.89	Female	CGA	1	7	0	C2	High-18-AS
S76	mRNA	45.89	Female	CGA	1	7	0	C1	Low-18-AS
S77	mRNA	17.96	Female	CGA	1	6	0	C1	Low-18-AS

S78	mRNA	25.18	Male	CGA	1	6	0	C1	Low-18-AS
S79	mRNA	28.13	Female	CGA	1	9	1	C1	High-18-AS
S8	mRNA	35.8	Male	CGA	0	34	1	C1	High-18-AS
S80	mRNA	57.65	Female	CGA	1	7	0	C1	Low-18-AS
S81	mRNA	19.34	Male	CGA	1	9	0	C1	Low-18-AS
S82	mRNA	17.6	Male	CGA	1	7	0	C1	Low-18-AS
S83	mRNA	5.25	Female	CGA	1	8	1	C3	High-18-AS
S84	mRNA	4.7	Male	CGA	1	16	1	C3	High-18-AS
S85	mRNA	9.18	Male	CGA	1	18	0	C3	High-18-AS
S86	mRNA	7.85	Male	CGA	1	16	1	C3	High-18-AS
S88	mRNA	3.58	Male	CGA	1	17	1	C2	High-18-AS
S89	mRNA	6.94	Male	CGA	1	10	1	C3	High-18-AS
S9	mRNA	21.64	Female	CGA	1	51	0	C2	High-18-AS
S90	mRNA	2.3	Female	CGA	1	60	0	C1	Low-18-AS
S91	mRNA	4.02	Male	CGA	1	60	0	C1	Low-18-AS
S92	mRNA	16.17	Male	CGA	1	60	0	C1	Low-18-AS
S93	mRNA	15.1	Female	CGA	1	15	1	C1	High-18-AS
S94	mRNA	2.76	Male	CGA	1	22.73333333	0	C1	Low-18-AS
S95	mRNA	2.06	Male	CGA	1	24.9	0	C1	Low-18-AS
S96	mRNA	2.8	Male	CGA	1	25.2	0	C1	High-18-AS
S97	mRNA	16.35	Male	CGA	1	15	0	C1	Low-18-AS
S98	mRNA	14.65	Female	CGA	1	9	1	C2	High-18-AS
S99	mRNA	15.56	Male	CGA	1	11	1	C2	High-18-AS

Table S2.Candidate drugs for CLUE analysis

id	pert_id	pert_iname	cell_iname	pert_type	moa	target_name
AICHI001_NALM6_24H:F19	BRD-K13646352	midostaurin	NALM6	trt_cp	FLT3 inhibitor|KIT inhibitor|PKC inhibitor	FLT3|KIT|CCNB1|FLT1|KDR|PDGFRB|PRKCA|VEGFA|PRKCG
AICHI001_NALM6_24H:B13	BRD-K85402309	dovitinib	NALM6	trt_cp	EGFR inhibitor|FGFR inhibitor|FLT3 inhibitor|PDGFR inhibitor|VEGFR inhibitor	FGFR1|FLT1|KDR|EGFR|FGFR3|FLT3|FLT4|KIT|PDGFRB|CSF1R|FGFR2|INSR|PDGFRA
EMU001_NALM6_24H:I19	BRD-K49328571	dasatinib	NALM6	trt_cp	KIT inhibitor|Bcr-Abl inhibitor|Ephrin inhibitor|PDGFR inhibitor|Src inhibitor|Tyrosine kinase inhibitor	ABL1|FYN|LCK|SRC|KIT|YES1|EPHA2|LYN|PDGFRB|BCR|BTK|DDR1|DDR2|PDGFRA|ABL2|BLK|FGR|FRK|HCK|SRMS|STAT5B
EMU001_NALM6_24H:M19	BRD-K19540840	saracatinib	NALM6	trt_cp	Src inhibitor	SRC|ABL1|LCK|YES1

TableS3. Details of RT-PCR primers used in this study

AS events	Forward Primer (5’-3’)	Reverse Primer (5’-3’)
BASP1-AS1_AL	GTAGCAACCCTTCTTTGGAT	CCATCTCAGCCTCAAGACT
FOXP1_AL	ATTTACAAGCAACACATGACAG	AGGCATGAAAATAGAGTTTCAGG
IRF4_SE	GTACTTAGGAGTTTAGCTTAAGTCG	CAAAAAACAAAAACGCCTTAATGTTC
LMBRD1_AF	GCCCCTGACATATGTGTCC	ACAGAACGTTTGAAAAAAGCTTC
LSS_A5	GAAAACATTGCTGGGGTCTTC	TGGAACGCACAGACGGCA
NUMB_AL	GGACCGAGTGGGGTCAATC	TTAAAGTTCAATTTCAAACGTCTTCTG
PDIA3_AF	GCGCAAGCAGCGGGTTAGT	CAGGGGGCGAAGAACTCGAC
EVL_AL	CCAGGAAAACTGAGCCCCA	TTGAAAAACTGGGAAAACATTTATTATCC
